# Characterizing autism spectrum disorders by key biochemical pathways

**DOI:** 10.3389/fnins.2015.00313

**Published:** 2015-09-24

**Authors:** Megha Subramanian, Christina K. Timmerman, Joshua L. Schwartz, Daniel L. Pham, Mollie K. Meffert

**Affiliations:** ^1^Solomon H. Snyder Department of Neuroscience, Johns Hopkins University School of MedicineBaltimore, MD, USA; ^2^Department of Biological Chemistry, Johns Hopkins University School of MedicineBaltimore, MD, USA

**Keywords:** autism spectrum disorders (ASD), neurotrophic factors, mTOR pathway, ERK signaling, MAP kinase signaling system, protein synthesis

## Abstract

The genetic and phenotypic heterogeneity of autism spectrum disorders (ASD) presents a substantial challenge for diagnosis, classification, research, and treatment. Investigations into the underlying molecular etiology of ASD have often yielded mixed and at times opposing findings. Defining the molecular and biochemical underpinnings of heterogeneity in ASD is crucial to our understanding of the pathophysiological development of the disorder, and has the potential to assist in diagnosis and the rational design of clinical trials. In this review, we propose that genetically diverse forms of ASD may be usefully parsed into entities resulting from converse patterns of growth regulation at the molecular level, which lead to the correlates of general synaptic and neural overgrowth or undergrowth. Abnormal brain growth during development is a characteristic feature that has been observed both in children with autism and in mouse models of autism. We review evidence from syndromic and non-syndromic ASD to suggest that entities currently classified as autism may fundamentally differ by underlying pro- or anti-growth abnormalities in key biochemical pathways, giving rise to either excessive or reduced synaptic connectivity in affected brain regions. We posit that this classification strategy has the potential not only to aid research efforts, but also to ultimately facilitate early diagnosis and direct appropriate therapeutic interventions.

## Introduction

Autism spectrum disorders (ASD) are a group of neurodevelopmental disorders frequently characterized by impairments in social interactions, difficulties with language and communication, and the presence of repetitive, perseverative behaviors (Abrahams and Geschwind, 2008; Zoghbi and Bear, 2012). While ASD are generally highly heritable and can be defined by symptoms in core areas, there exists significant heterogeneity in genetics, phenotypes, clinical presentation, and associated comorbidities (Persico and Bourgeron, 2006). Recent advances have identified hundreds of genetic risk factors, including common and rare genetic variants, which can increase the likelihood of ASD (Ronemus et al., 2014). Many autism susceptibility genes are known to have important roles in brain development, with functions ranging from synaptic transmission to RNA processing and neurogenesis (Gilman et al., 2011; O'Roak et al., 2012; De Rubeis et al., 2014). However, the plethora of genetic targets has highlighted the need for the ASD research community to understand whether genes implicated in ASD may converge on common cellular and developmental processes that can ultimately disrupt functions of brain circuits mediating language, cognition, and social behavior.

Attempts have been previously made to stratify ASD patients into smaller, more homogeneous subgroups by utilizing specific genetic signatures (Bernier et al., 2014) or behavioral and clinical endophenotypes (Spence et al., 2006; Eapen and Clarke, 2014). However, these strategies face difficulty encompassing the genetic and phenotypic heterogeneity of ASD, and may not assist in the identification of common neurobiological pathways underlying disease. In this review, we propose that genetically diverse forms of ASD may be usefully parsed into entities resulting generally from either synaptic and neural overgrowth or undergrowth, and the corresponding alterations in key biochemical pathways supporting these phenotypes. We review recent studies in patients and mouse models of ASD indicating convergence toward these two fundamental biological processes among genetically diverse causes of autism. We also discuss potential molecular signaling pathways that may contribute to these general alterations in growth and neural connectivity in ASD. The review primarily emphasizes data reported from earlier developmental stages to maintain a focus on alterations that are potentially causal, rather than secondary results of ASD pathology.

**Figure 1:**
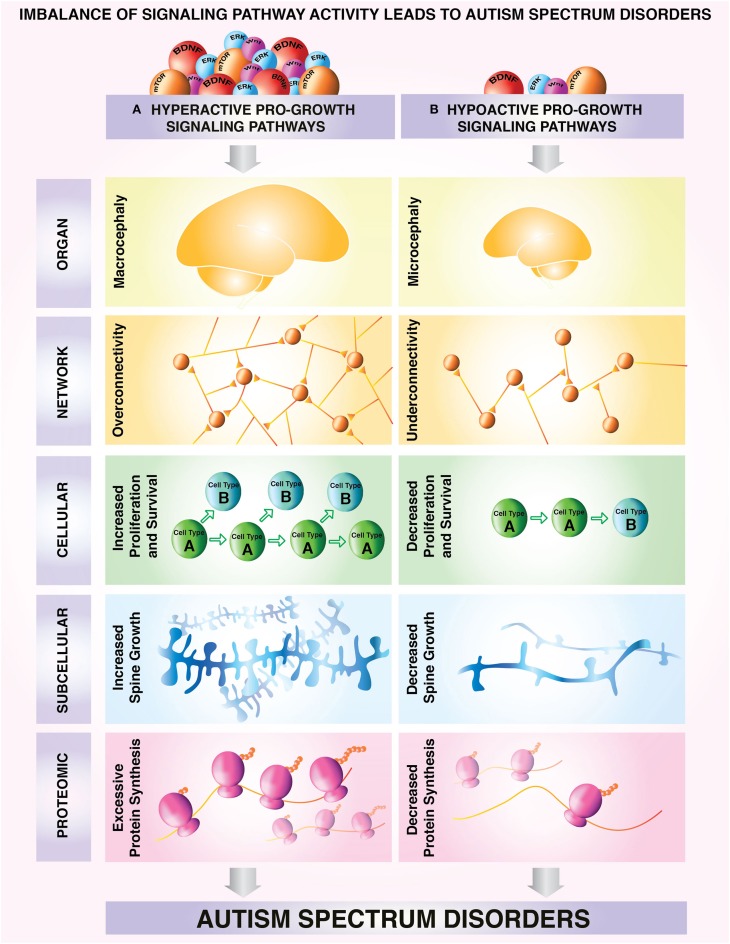
**Model depicting a proposed classification of different subtypes of ASD based on correlates of growth state**. Neural overgrowth and undergrowth phenotypes have been associated with aberrant regulation of growth control pathways in autism spectrum disorders. Characteristic overgrowth and undergrowth phenotypes can be observed consistently from molecular to cellular and network levels. **(A)** Upregulation of pro-growth pathways can lead to: macrocephaly (Courchesne et al., 2003, 2007) aberrant overconnectivity of neuronal networks (Meikle et al., 2007; Keown et al., 2013; Supekar et al., 2013) increased survival and proliferation at the cellular level (Castrén et al., 2005; Callan et al., 2010) increased synaptic growth at the subcellular level (Irwin et al., 2001; Jaworski et al., 2005; Kwon et al., 2006; Tang et al., 2014), excessive protein synthesis (Osterweil et al., 2013; Santini et al., 2013) and/or a selective protein synthesis program enhancing production of growth-promoting proteins. **(B)** In contrast, hypoactive growth pathways lead to microcephaly, (Bronicki et al., 2015; Van Bon et al., 2015) underconnectivity of neuronal networks (Assaf et al., 2010; Anderson, 2014) decreased survival and proliferation at the cellular level (Yufune et al., 2015), decreased synaptic growth at the subcellular level (Cheng et al., 2014), decreased protein synthesis (Li et al., 2013; Tian et al., 2015) and/or a protein synthesis program that does not promote growth. These example phenotypes of undergrowth and overgrowth can be used as readouts for categorization of growth status in autism spectrum disorders.

We propose that stratifying ASD based on readouts of over- or under-growth is an informative approach for establishing a more homogeneous sample. This will facilitate future research into the mechanisms underlying autism pathogenesis. This approach also has the potential to enable autism risk assessment, accelerate progress in pre-clinical research and clinical trials to facilitate an individualized approach to treatment, and avoid the discarding of potentially useful therapeutic strategies. Figure 1 organizes over- and under-growth phenotypes seen in ASD models and patients, and pairs reviews and primary literature examining these phenotypes.

## Brain growth, structure, and connectivity in ASD

One of the earliest indications of aberrant brain growth during development in ASD came from measurements of head circumference among infants and young children with autism. Head circumference is posited as a reliable proxy for relative brain size during early postnatal ages (Bartholomeusz et al., 2002). These studies have provided important initial evidence for the presence of both over- and under-growth in ASD. Many studies have shown head circumference to be abnormally enlarged in children with ASD around the age of symptomatic diagnosis (Courchesne et al., 2003, 2007). A study examining 126 autistic children (2–16 years of age) reported a higher than expected incidence of both macrocephaly and microcephaly (Fombonne et al., 1999).

Reciprocal changes in growth as grossly measured by head size have also emerged as characteristic features of an increasing number of syndromic and non-syndromic forms of ASD. For example, de novo mutations in the dual-specificity tyrosine-(Y)-phosphorylation-regulated kinase 1 A (DYRK1A) gene are associated with a type of syndromic ASD and intellectual disability that presents with microcephaly (Bronicki et al., 2015; Van Bon et al., 2015). Conversely, macrocephaly has been shown to occur in a subset of ASD patients harboring disruptive mutations in the ASD-linked chromodomain helicase DNA binding protein 8 (Chd8) gene or deletions in 17q12 (Moreno-De-Luca et al., 2010; Bernier et al., 2014). This phenotype is further discussed in the context of specific, genetically defined causes of ASD later in this review. It is important to note that this early pathology of dysregulated brain growth tends to normalize later in childhood and adolescence, highlighting the necessity to focus on alterations that occur during the critical periods of prenatal and early postnatal development in ASD.

The application of neuroimaging methods to the study of ASD has provided unprecedented insights into the structure and intrinsic connectivity patterns of brain regions involved in complex social behavior and cognition. Due to technical constraints, previous assessments of functional brain connectivity in ASD were limited to task-based activation studies in adults with high-functioning autism, with relatively small sample sizes and varied methodologies (reviewed in Müller et al., 2011; Vissers et al., 2012). Brain changes observed in adulthood, however, may not reflect primary aberrations and may not generalize to children with severe low-functioning autism. Notably, it is now also understood that lowered task-based activation in fMRI could reflect cognitive performance deficits, rather than an intrinsic property of brain function. Recent studies have provided some clarification by taking advantage of technical advancements in imaging and analysis to conduct resting-state fMRI (R-fMRI) studies in younger ASD subject populations in order to provide a more accurate model of the developmental origins of the disease. These studies are useful for an initial assessment of the feasibility of categorizing ASD by over- or under-growth.

Supekar et al. (2013) and Keown et al. (2013) focused their R-fMRI analyses to young children and adolescents with ASD (7–14 years) to show increased long-range and short-range intrinsic connectivity across multiple brain regions. Moreover, the degree of functional hyperconnectivity was positively correlated with severity of social and repetitive behavioral symptoms (Keown et al., 2013; Supekar et al., 2013). Conversely, decreased functional connectivity has been observed in long-range interhemispheric projections and default mode networks that typically exhibit greater activity under resting conditions as opposed to tasks that require significant attention (Assaf et al., 2010; Anderson et al., 2011; Anderson, 2014). In certain cases, decreased functional connectivity has been inversely correlated to severity of core behavioral deficits in ASD (Assaf et al., 2010). Collectively, the fact that reciprocal changes in the intrinsic functional architecture of the brain may result in phenotypic outcomes associated with ASD supports the involvement of both under- and over-growth mechanisms in ASD pathophysiology.

Recent work by Ellegood et al. has shown that mouse models of ASD may be clustered in broad categories of brain under- and over-connectivity, providing further support for parsing the heterogeneity of ASD using this approach (Ellegood et al., 2015). In patients, the majority of existing structural and neuroimaging studies have focused on delineating the effects of age on intrinsic connectivity patterns. As a result, while this approach holds promise, we must await further work in order to assess whether different genetic causes of autism may also classify into over- or under-growth based on structural and functional measures of brain connectivity.

## Molecular readouts of growth

Mounting evidence has highlighted key growth signaling pathways that are frequently perturbed in patients with ASD as well as mouse models of autism. This section will cover these key molecular readouts of growth in autism, including dysregulated growth factor, mammalian target of rapamycin (mTOR), and extracellular signal-regulated kinase (ERK1/2) signaling. Characterizing how the regulators and downstream correlates of these growth pathways are dysregulated in ASD will allow us to better classify forms of autism into broad, but more homogeneous subtypes. This knowledge will be valuable for (i) diagnosis of under- or over-growth phenotypic subtypes of ASD, (ii) selection of homogeneous patient populations for more rigorously controlled studies, and (iii) a potential biomarker that can be used to potentially assess efficacy of clinical trials. We propose that broadly stratifying patient populations in terms of under- or over-growth phenotypes based on the following signaling pathways would facilitate mechanistic and therapeutic insights into ASD, without necessitating an impractical fine categorization by precise genetic etiology.

### Growth factor signaling

Neurotrophins, such as nerve growth factor (NGF) and brain-derived neurotrophic factor (BDNF) were first identified as target-derived survival factors. However, growing body of evidence has indicated that neurotrophins regulate many aspects of neuronal structure and function, including neurodevelopment, differentiation, morphogenesis, and synaptic plasticity (Poo, 2001; Reichardt, 2006). Subsequent research over the past half century has revealed the existence of other canonical neurotrophins, including neurotrophin-3 (NT-3), and neurotrophin-4 (NT-4). Each of the four mammalian neurotrophins binds to and activates one or more of the three members of the tropomyosin-related kinase (Trk) family of receptor tyrosine kinases (TrkA, TrkB, and TrkC), leading to subsequent activation of phosphatidylinositol 3-kinase (PI3K), phospholipase C (PLC), ERK1/2, and mTOR signaling (Chao et al., 2006; Reichardt, 2006). Additionally, appropriate control of neurotrophin signaling at multiple regulatory levels (epigenetic, transcriptional, post-transcriptional, and post-translational) is critical to physiological functions, such as cell fate decisions, axon growth, and dendritic growth and pruning, and overall neuronal network connectivity (Reichardt, 2006; Park and Poo, 2013). Genetic knockout strategies and pharmacological interventions reveal that many growth regulatory effects of neurotrophins depend upon Trk signaling pathways and subsequent activation of downstream cellular cascades (Reichardt, 2006; Park and Poo, 2013). Consistent with their potent roles as regulators of neuronal proliferation, survival, differentiation, and morphogenesis, dysregulated neurotrophin signaling has been implicated in neurodegenerative disorders, such as Alzheimer's and Huntington's disease, and also psychiatric disorders, including depression, substance abuse, as well as autism (Tsai, 2005; Chao et al., 2006; Martinowich et al., 2007; Nishimura et al., 2007; Gadow et al., 2009; Sadakata and Furuichi, 2009).

Other trophic factors, such as glial-derived neurotrophic factor (GDNF), vascular endothelial growth factor (VEGF), and ciliary neurotrophic factor (CNTF), as well as insulin-like growth factor (IGF) also induce pro-growth signaling pathways. Like BDNF, NGF, NT-3, and NT-4, these additional trophic factors also signal through receptor tyrosine kinases to elicit downstream signaling cascades that promote growth in a variety of tissues, including neurons (Junger and Junger, 1998; Ferrara et al., 2003; Chao et al., 2006; Reichardt, 2006; Laviola et al., 2007). Dysregulation in these trophic factors has been documented in ASD, and extensive work in neurodegenerative disorders has characterized their trophic and pro-survival effects in the brain, including in motor neuron atrophy (IGF and CNTF), neuropathies of the peripheral nervous system (NGF and NT-3), Alzheimer's disease (NGF, BDNF, IGF), diabetic retinopathy (CNTF, BDNF), Huntington's disease (BDNF, NT-3, NT-4), Parkinson's disease (BDNF, GDNF), and amyotrophic lateral sclerosis (VEGF and IGF) (Hefti, 1994; Dawbarn and Allen, 2003; Gasparini and Xu, 2003; Zuccato and Cattaneo, 2009; Weissmiller and Wu, 2012; Ola et al., 2013; Keifer et al., 2014). The pronounced pro-growth and proliferative effects of these neurotrophins and their downstream effectors, such as mTOR and ERK1/2, make dysregulation in trophic factor cascades excellent readouts for stratification of ASD into generally overgrowth or undergrowth entities.

### mTOR

mTOR is a highly conserved and ubiquitously expressed serine/threonine kinase that serves as an important regulator of cellular growth, metabolism, and survival in both developmental and disease states across a variety of tissue types. mTOR functions in two heteromeric and functionally distinct protein complexes, mTORC1 and mTORC2, which are embedded into complex signaling networks. In the brain, mTOR integrates inputs from a range of extracellular sources, including growth factors, guidance cues, and nutrients (Takei and Nawa, 2014).

mTOR regulates many processes that are essential for growth by serving as a nexus for controlling protein synthesis, energy homeostasis, metabolism and actin cytoskeletal dynamics. Studies of tumorigenesis strongly implicate mTOR activity as a correlate of growth and metabolic status. Many familial cancer syndromes result from mutations in genes encoding upstream proteins that influence mTOR activation, including Tsc1/2, PTEN, and neurofibromatosis type I (NF1) (Yuan and Cantley, 2008). Dysregulation of genes in this pathway has also been linked to multiple disease conditions, including ASD, type II diabetes, obesity, and neurodegeneration (Zoncu et al., 2011; Laplante and Sabatini, 2012; Takei and Nawa, 2014).

Importantly, bidirectional changes in mTOR signaling have also been shown to result in opposing downstream effects on neuronal growth and morphogenesis. In mice, ablation of mTOR or associated components that regulate mTORC1/2 assembly and signaling leads to embryonic lethality (Guertin et al., 2006; Shiota et al., 2006). Conditional deletion of both mTORC1 and mTORC2 in neural progenitors of the developing CNS can cause microcephaly due to an overall decrease in neuronal number and size as a result of reduced neuronal progenitor proliferation and suppressed differentiation of cortical neurons (Cloëtta et al., 2013; Thomanetz et al., 2013; Ka et al., 2014). Conversely, enhanced mTOR signaling following inactivation of upstream negative regulators (PTEN and TSC1/2), or constitutive activation of positive regulators (PI3K, Akt, and Ras), has been associated with macrocephaly, neuronal hypertrophy, and increased soma size and dendritic complexity of hippocampal neurons (Jaworski et al., 2005; Kwon et al., 2006). These effects of mTOR activation on neuronal and dendritic morphogenesis requires novel protein synthesis (Jaworski et al., 2005).

mTOR-mediated regulation of protein translation has garnered much interest in the field of ASD research. Substrates of mTOR that are critically involved in the translation initiation machinery, such as p70 ribosomal S6 kinase 1 (S6K1) and the eukaryotic translation initiation factor 4E-binding proteins (4E-BPs), are emerging as key players in autism pathogenesis (Klann and Dever, 2004). Phosphorylation of S6K1 by mTORC1 promotes ribosomal biogenesis, translational initiation, and elongation through a variety of effectors (Ma and Blenis, 2009). Mice harboring a genetic deletion of S6K1 are significantly smaller than wild-type counterparts. Certain protein synthesis-dependent forms of synaptic plasticity which may represent correlates of growth in adulthood, such as mGluR-LTD, have been associated with increased phosphorylation and activation of S6K1 (Antion et al., 2008).

Unphosphorylated 4E-BP2 inhibits cap-dependent protein synthesis by sequestering the translation initiation factor eIF4E. Phosphorylation of 4E-BP2 by mTORC1 leads to its dissociation from eIF4E, thereby de-repressing its cap-binding activity and enabling formation of the eIF4F translation initiation complex. Deletion of 4E-BP2 or overexpression of eIF4E *in vivo* leads to increased eIF4F complex formation, facilitated protein synthesis-dependent long-term synaptic plasticity, elevated dendritic spine density, and behavioral abnormalities reminiscent of ASD (Banko et al., 2005, 2006; Gkogkas et al., 2013; Santini et al., 2013).

Taken together, bidirectional changes in mTOR signaling lead to opposite effects on protein synthesis, metabolism, and growth. Therefore, measuring activity levels of mTOR or downstream effectors of this cascade may serve as reliable indicators of altered pro- or anti-growth states in the brain.

### ERK1/2

ERK1/2 are paralogous members of the MAPK signaling cascade with well-characterized roles in regulating growth at cellular and organismal levels. The canonical MAPK/ERK pathway transduces signals from cell surface receptors to the nucleus through sequential phosphorylation steps. This pathway is responsive to growth factors, chemokines, oxidative stress, and cytokines (Lu et al., 2005; Byts et al., 2006; Samuels et al., 2009; Hsieh et al., 2010). The ERK1/2 signaling cascade has important roles in cellular proliferation, differentiation, and apoptosis (Murphy and Blenis, 2006). In the nervous system, activation of this pathway generally promotes excitation and is involved in activity-dependent plasticity, long-term potentiation (LTP), long term depression (LTD) and memory formation (Satoh et al., 2011). Downstream targets of ERK1/2 signaling in neurons govern processes such as dendritic spine stabilization, modulation of ion changes and receptor insertion (Sweatt, 2004).

Recent genome-wide association studies (GWAS) and analysis of copy number variations (CNV) have identified an enrichment of MAPK/ERK signaling components in patients with autism (Pinto et al., 2010). Furthermore, ERK1/2 activation is required for the formation and stabilization of dendritic spines (Wu et al., 2001; Goldin and Segal, 2003) and activation of ERK1/2 by growth factors has well-characterized functions in regulating cell proliferation in the CNS (Sweatt, 2004; Samuels et al., 2009). ERK1/2 plays a critical role in corticogenesis through regulation of the cell cycle in proliferation of neural progenitor cells. While both ERK1 and ERK2 are expressed highly in the adult brain, ERK2 levels are higher than ERK1 (Samuels et al., 2009). Additionally, only loss of ERK2 results in early embryonic lethality in mouse models primarily through a failure of normal placental and trophoblast development (Samuels et al., 2009). Conditional knockout of ERK2 during the height of cortical neurogenesis results in a decrease in neuron number and subsequent increases in astrocyte number in the murine cortex. This decrease reflects alterations in growth and proliferation by a reduction in the number of cell divisions in intermediate progenitor cells leading to decreased cortical thickness (Samuels et al., 2008).

The strongest evidence implicating disruption of ERK1/2 signaling in ASD comes from a number of single gene mutations that are associated with autism including Tuberous sclerosis, Fragile X syndrome, 16p11.2 (discussed in future sections in this review) and NF1 all ultimately leading to activation of ERK1/2. NF1 is a GTPase-activating protein (GAP) for Ras, an upstream activator of ERK1/2. Mutations in NF1 cause neurofibromatosis type 1, an autosomal inherited disorder with a high frequency of hyper-proliferative schwannoma cancers. More than 50% of individuals with mutations in NF1 also have cognitive impairments and recent studies report a significant increase in the incidence of ASD in NF1 patients (Marui et al., 2004). In addition to the numerous monogenic forms of autism implicating ERK1/2 activity in disease pathophysiology, the inbred mouse strain BTBR, a model of non-syndromic autism, shows an increase in phospho-ERK1/2 levels in the pre-frontal cortex (Faridar et al., 2014).

ERK1/2 is a critical regulator of development and alterations in ERK1/2 activity, either increased active phospho-ERK1/2 or decreased phospho-ERK1/2, have been associated with autistic features (Wang et al., 2012; Yufune et al., 2015). Interestingly, a recent study conducted in mice suggests that there may be a critical window for alterations in ERK1/2 activity to lead to the development of ASD. The authors find that blockade of ERK1/2 signaling through administration of the MEK inhibitor SL327 at postnatal day 6 leads to adult mice exhibiting common autistic behavioral phenotypes, such as deficits in social interaction, as well as increased apoptosis in the forebrain (Yufune et al., 2015). However, inhibition of ERK1/2 at postnatal day 14 did not lead to the development of autistic behaviors in these mice, suggesting a critical window in development for regulation of ERK1/2 activity. Collectively, strong evidence from multiple biological settings links ERK1/2 pathways to the regulation of growth states, including proliferation and excitation.

## Characterization of genetically defined models of ASD based on growth readouts

This section reviews ASD of known genetic cause and the mouse models of these ASD with reference to growth pathway correlates (Table 1). The presented mouse models recapitulate many phenotypes of human ASD, including dysregulation of neuronal growth and development, synaptic transmission, neuronal connectivity, and behavior. Although each mouse model does not perfectly genocopy the human genetic variants that predispose individuals to ASD, they present valuable resources for mechanistic investigations into the genetic pathways underlying aberrant neuronal growth. Moreover, given that several of these models involve copy number variants (CNVs) as well as deletions and duplications of a given chromosomal locus, these models provide insights into how gain or loss-of-function can impact the spectrum of neuronal growth phenotypes inherent in patients with ASD. Mouse models of ASD of known genetic etiology will be informative for diagnosis and future clinical studies that target pathways of cellular growth that are dysregulated in individuals with ASD.

**Table 1:** **Classification of well-established monogenic and copy number variant models of ASD based on correlates of neuronal growth and connectivity**.

**Genetic etiology**	**Associated syndrome**	**Synaptic or neuronal growth phenotype**
Fmr1	Fragile X syndrome	Overgrowth
TSC	Tuberous sclerosis	Overgrowth
PTEN	N/A	Overgrowth
MeCP2 loss-of-function	Rett syndrome	Undergrowth
MeCP2 gain-of-function	MeCP2 duplication syndrome	Overgrowth
AUTS2	Syndromic intellectual disability	Undergrowth
16p11.2 microdeletion	Non-syndromic ASD	Overgrowth
16p11.2 duplication	Non-syndromic ASD	Undergrowth
22q11.2 microdeletion	Non-syndromic ASD	Undergrowth

## Monogenic models of ASD

### Fragile X syndrome

Fragile X syndrome (FXS) is the most common inherited cause of ASD and intellectual disability, affecting approximately 1 in 4000 males and 1 in 8000 females worldwide (Peprah, 2012). Accounting for 2–6% of all cases of autism, FXS is a neurodevelopmental disorder that is associated with an expansion in a CGG trinucleotide repeat element in the 5′ UTR of the Fragile X Mental Retardation 1 (FMR1) gene. The mutation leads to hypermethylation of the locus and transcriptional silencing of FMR1, which subsequently leads to a loss of production of its downstream gene product, Fragile X Mental Retardation Protein (FMRP) (Penagarikano et al., 2007). FMRP is localized in the soma and dendrites of neurons, where it functions predominantly to suppress the translation of a subset of mRNAs (Penagarikano et al., 2007). The anatomical, synaptic, and molecular features of FXS have made this monogenic disorder a classic model of developmental overgrowth in ASD.

FXS patients display intellectual disability and cognitive impairments, developmental delay and physical features consistent with overgrowth, such as macrocephaly, elongated facial morphology, and enlarged ears (Chudley and Hagerman, 1987). Additional growth deficits include an increase in body weight in a subset of FXS patients (Nowicki et al., 2007) and macroorchidism, or enlarged testicles, in males following the onset of puberty (Chudley and Hagerman, 1987). In postmortem brain tissue isolated from FXS patients, studies have found an overall increase in dendritic spine density as well as more immature spine morphology (Irwin et al., 2001). FXS patients and mouse models deficient in FMRP also display hallmarks of brain morphology and function found in other genetic causes of autism defined by overgrowth. These include an early overabundance of synapses, brain hyperconnectivity, and excessive basal and activity-responsive neuronal protein synthesis (Dölen et al., 2007; Gibson et al., 2008; Pan et al., 2010).

In the central nervous system and the testis, two tissues in which FMRP is known to be highly expressed (Penagarikano et al., 2007), loss of FMRP function causes gross morphological abnormalities. For example, children with FXS and mice deficient in FMRP display significantly increased hippocampal volume (Kates et al., 1997; Shi et al., 2012). Specifically, structural MRI methods show an increase in hippocampal volume from P18–30 in Fmr1 KO mice (Shi et al., 2012). Another well-described and reproducible growth defect observed in patients and mouse models of FXS is macroorchidism. Fmr1 KO mice display an elevation in testes weight that has been linked to increased proliferation of supporting Sertoli cells during embryonic development (Slegtenhorst-Eegdeman et al., 1998; Dölen et al., 2007).

Accumulating reports further indicate a critical role for FMRP function in the proper control of developmental timing. FMRP deficiency leads to temporal delays in many developmental settings, including the perinatal critical period for barrel cortex plasticity (Harlow et al., 2010) and the switch in polarity of GABA signals from depolarizing to hyperpolarizing (He et al., 2014). Loss of FMRP also causes increased proliferation and abnormal differentiation of neural stem and progenitor cells (Castrén et al., 2005; Callan et al., 2010).

Abnormalities in dendritic spines are thought to be a central feature of FXS, but the precise nature of the defect is still controversial. Although there are several reports of altered spine density and morphology in the Fmr1 KO mouse model, the observed alterations appear to vary considerably depending on the culture/staining methods, developmental age, brain region, and mouse background strain (He and Portera-Cailliau, 2013). More recent studies using *in vivo* two-photon microscopy to image dendritic spine dynamics in intact neocortex from Fmr1 KO mice have revealed specific defects in spine turnover and maturation that reflect a pro-growth state during development. Cortical pyramidal neurons display abnormally high rates of spine turnover and delayed stabilization of spines during early postnatal development, coinciding with the critical period for spine formation and plasticity in the neocortex (Cruz-Martín et al., 2010; Pan et al., 2010).

At the functional level, Fmr1 KO mice exhibit network hyperexcitability and an imbalance between excitation and inhibition in neural circuits (Gibson et al., 2008; Gonçalves et al., 2013). While excessive neuronal growth and delayed synaptic maturation during critical periods for experience-dependent plasticity in the brain can influence the wiring and functional integrity of neural circuits, the exact outcome of such changes are likely mediated in a cell type-specific manner. Therefore, future studies need to examine molecular readouts of growth in different cell types in the brain, and in the context of specific functional circuits.

Several studies using neuronal and peripheral tissues from FXS patients and Fmr1 KO mice have investigated ERK1/2 phosphorylation status. Although results from these studies have shown some discrepant findings, possibly due to the labile nature of ERK1/2 phosphorylation, a number of reports have shown elevated basal phospho-ERK1/2 (pERK1/2) levels in brain tissues from Fmr1 KO mice and FXS patients (Michalon et al., 2012; Wang et al., 2012). Inhibition of the ERK1/2 pathway normalizes excessive hippocampal protein synthesis and also alleviates certain behavioral abnormalities observed in Fmr1 KO mice, including audiogenic seizure susceptibility (Wang et al., 2012; Osterweil et al., 2013). Further, a phase I clinical trial of the Ras-ERK1/2 inhibitor, lovastatin, conducted by Dr. Francois Corbin's group showed significant behavioral improvements in FXS patients (Çaku et al., 2014).

In addition to hyperactive ERK1/2, another critical molecular player that may contribute to pro-growth phenotypes in FXS is dysregulated mTOR signaling. Although most analyses so far have been conducted at older ages, elevated levels of phospho-AKT, phospho-S6K, and active phospho-eIF4E have been detected in brain tissue and peripheral blood lymphocytes from FXS patients (Hoeffer et al., 2012). Similar hyperactivation of several upstream and downstream components of the mTOR cascade have been observed in brains of Fmr1 KO mice (Sharma et al., 2010). Additionally, deletion of S6K in Fmr1 KO mice rescues exaggerated hippocampal protein translation, aberrant dendritic spine morphology and macroorchidism (Bhattacharya et al., 2012). Normalizing elevated levels of PI3K enhancer (PIKE), the upstream activator of PI3K-Akt-mTOR signaling, in Fmr1 KO mice also normalizes dendritic spine density and network hyperexcitability (Gross et al., 2015).

Hyperactivation of the ERK1/2 and mTOR pathways in FXS has been generally linked to excessive global protein synthesis in the brain. Auerbach et al. elegantly demonstrated that restoring the optimal balance of intracellular signaling and downstream protein synthesis may reverse synaptic and behavioral defects in mouse models of FXS and TSC (Auerbach et al., 2011; Bhakar et al., 2012). Changes in protein synthesis have become a focal point in the study of ASD, and are one of multiple key cellular readouts for growth state. It is worth noting, however, that global upregulation or downregulation of mRNA translation may not directly result in overgrowth or undergrowth phenotypes, which are likely to depend upon the identity of the affected mRNAs. For example, an overgrowth phenotype might also be achieved by an alteration in the specificity of protein synthesis to increase translation of pro-growth genes without any change in total protein synthesis. The regulation of gene target selectivity in translation may also play a role in the contribution of altered protein synthesis to pro- or anti-growth phenotypes observed in subtypes of ASD.

There is also evidence supporting dysregulation of upstream activators of the ERK1/2 and mTOR pathways in autism, including BDNF signaling through the TrkB receptor (Maija Castrén and Castrén, 2014). *TrkB* mRNA has been identified as a target for translational suppression by FMRP (Darnell et al., 2011). While it is clear that BDNF/TrkB signaling is altered in FXS, the precise effects of FMRP loss on spatiotemporal expression patterns or activity of BDNF and the TrkB receptors are currently unknown. Elevated catalytic TrkB expression has been observed in undifferentiated neural progenitor cells from Fmr1 KO mice (Louhivuori et al., 2011). BDNF expression in the hippocampus varies with age in Fmr1 KO mice, with levels significantly higher than wild-type controls at 2 months of age (Uutela et al., 2012). However, alterations in BDNF expression appear to differ by brain region (Louhivuori et al., 2011). Since BDNF is known to establish pro-growth programs of gene expression that are important for neuronal proliferation and morphogenesis, elevations in BDNF signaling may significantly contribute to hypertrophic phenotypes observed in FXS (Poo, 2001; Reichardt, 2006).

Another line of evidence pointing to upregulated BDNF signaling in FXS comes from recent studies demonstrating aberrant increases in matrix metalloprotease-9 (MMP9) levels in brains of FXS patients and Fmr1 KO mice (Gkogkas et al., 2014). Elevated protein levels of MMP9 have also been detected in plasma and amniotic fluid derived from FXS and ASD patients (Dziembowska et al., 2013; Leigh et al., 2013). MMP9 is a protease that can cleave pro-BDNF to mature BDNF in the hippocampus (Mizoguchi et al., 2011) and developing neuromuscular junction (Je et al., 2012). Two studies demonstrated that genetic and pharmacological reduction of MMP9 levels ameliorates FXS-associated anatomical and behavioral abnormalities (Gkogkas et al., 2014; Sidhu et al., 2014). Treatment with a tetracycline derivative, minocycline, ameliorates enhanced MMP9 levels and significantly improves behavioral performance in Fmr1 KO mice (Bilousova et al., 2009). In clinical trials, treatment of FXS children and adults with minocycline has been found to be well-tolerated and results in significant behavioral improvements, as measured by the Clinical Global Impression Scale and ABC-C Irritability Subscale (Paribello et al., 2010; Leigh et al., 2013). Further, clinical responses to minocycline are correlated with changes in plasma MMP9 levels (Dziembowska et al., 2013). Together, these findings point to a potential involvement of the BDNF/MMP9 axis in FXS pathogenesis that would be worth exploring in future studies.

Collectively, work by several groups indicates that hyperactive ERK1/2, mTOR, and BDNF pathways may contribute to neuronal protein synthesis, growth, and connectivity defects in FXS. Therefore, the molecular and biochemical signatures of FXS are in accordance with its categorization as an overgrowth form of ASD.

### Tuberous sclerosis

Tuberous sclerosis (TSC) is a neurodevelopmental disorder that is caused by autosomal dominant mutations in the TSC1 or TSC2 tumor suppressor genes, which function as negative regulators of the mTOR signaling cascade. Loss of TSC1/2 function which enhances cell growth and promotes dysregulated metabolism leads to non-malignant tumor formation in the skin, brain, and other organs (Curatolo et al., 2008). Importantly, almost 50% of individuals with TSC also meet criteria for diagnosis of ASD or intellectual disability (Curatolo et al., 2008; Jeste et al., 2008). Most commonly used mouse models of TSC, such as mice harboring heterozygous genetic deletions of Tsc1 or Tsc2, exhibit phenotypes that recapitulate aspects of the human disease, including synaptic dysfunctions, deficits in learning and memory, and impaired social interactions (Goorden et al., 2007; Ehninger et al., 2008; Sato et al., 2012; Tang et al., 2014).

Homozygous deletion of Tsc1 in postnatal forebrain neurons using CaMKII-CRE Tsc1^flox/flox^ mice leads either to lethality or severe brain enlargement and neuronal hypertrophy in animals surviving to 3 months of age (Ehninger et al., 2008). Initial studies characterizing the function of TSC1/TSC2 complexes in the brain identified important roles in regulating neuronal growth and neural network homeostasis. In particular, complete *in vivo* loss of Tsc1 in post-mitotic neurons induces ectopic axon formation (Choi et al., 2008), enlarged and dysplastic neuronal morphology (Meikle et al., 2007), and network hyperexcitability due to weakened functional synaptic inhibition (Bateup et al., 2013). Recent work has also demonstrated that Tsc1 and P20-P30 Tsc2 deficient mice display reduced pruning of dendritic spines during a critical period in development, giving rise to increased spine density at later postnatal ages. Tang et al. attributed the observed failure of developmental spine pruning in mouse models of TSC to a deficit in mTOR-dependent macroautophagy (Tang et al., 2014).

Importantly, many of the aforementioned hypertrophic phenotypes of TSC loss-of-function are ameliorated by treatment with the mTORC1 inhibitor, rapamycin, suggesting that hyperactive mTOR signaling plays a critical role in neural overgrowth and hyperconnectivity defects in TSC. For most studies conducted, rescue of synaptic or behavioral abnormalities has been most successful if rapamycin is administered chronically beginning at early postnatal ages. This further supports the possibility that early mTOR hyperactivation may be the primary insult that triggers downstream effects on neuronal growth, development, and excitability. Although mTOR signaling appears to be persistently increased following TSC1/2 deficiency, secondary pathways may be engaged to maintain synaptic and behavioral defects into adulthood. Therefore, it remains to be assessed whether synaptic overgrowth and associated dysfunctions may be reversed by mTOR inhibition later in adulthood.

In accordance with known functions of mTOR in the regulation of mRNA translation, hyperactivation of this pathway would be expected to enhance global protein synthesis. Unexpectedly, adult Tsc2 haploinsufficient mice exhibit basal suppression of hippocampal protein synthesis (Auerbach et al., 2011). While examination of mRNA translation at early postnatal ages is required, the reported reduction in basal protein synthesis in adult Tsc2 deficient mice could reflect the tight homeostatic regulation of mTOR signaling. Work in non-neuronal systems has found that long-term hyperactivation of mTOR can evoke compensatory downregulation of upstream signaling factors through an inhibitory feedback loop (Hay, 2005). For example, murine embryonic fibroblast cultures derived from Tsc2 null mice exhibit increased mTOR activation, but reduced upstream PI3K-Akt signaling (Zhang et al., 2003). The contribution of developmental and homeostatic changes in growth pathway readouts further underscore the importance of focusing on early developmental ages for the classification of ASD subtypes.

Recent studies have also demonstrated that TSC1/2 may interact with the MAPK/ERK signaling cascade. This relationship was first appreciated in clinical studies which revealed that components of the ERK1/2 pathway are constitutively activated in TSC-associated brain lesions and tumor cell lines (Govindarajan et al., 2003; Ma et al., 2005, 2007). Upregulated ERK1/2 signaling has been observed in the ΔRG model of TSC, which recapitulates human deletion mutations that disrupt TSC1/2 complex function by interfering with Tsc2 GAP activity (Chévere-Torres et al., 2012). The authors of the same study reported no differences in ERK1/2 phosphorylation in hippocampi from Tsc1 (CamKII-CRE Tsc1^+/fl^) and Tsc2 (CamKII-CRE Tsc2^fl/fl^) mutant mice as compared to wild-type controls. However, this experiment was performed in adult animals where compensatory changes could have occurred, and therefore, more thorough analysis of phospho-ERK1/2 levels throughout development may be required.

TSC2 has, itself, also been implicated as a direct target of ERK1/2 kinase activity, wherein phosphorylation of TSC2 by ERK1/2 results in dissociation of the TSC1/2 tumor suppressor complex and subsequent disinhibition of mTOR-dependent effects on growth, proliferation, and protein synthesis (Ma et al., 2005). Although these studies were conducted in the context of tumorigenesis, this mechanism may be conserved in the nervous system as well. The fact that TSC1/2 serves as an important node of cross-talk and convergence between the ERK1/2 and mTOR signaling cascades might explain how changes in components of both pathways can produce a dysregulated growth state in many syndromic and non-syndromic forms of ASD. Based on the aforementioned phenotypes resulting from mutations in the TSC tumor suppressor, we propose that ASD patients with TSC loss-of-function represent an example characterized by neuronal overgrowth phenotypes.

### Phosphate and tensin homolog (PTEN)

The tumor suppressor gene PTEN is a dual protein and lipid phosphatase critical for modulating cellular growth, proliferation and survival. PTEN is a major inhibitor of the highly conserved PI3K-Akt signaling pathways, and regulates diverse cellular processes, including metabolism, survival, proliferation, growth, and motility (Worby and Dixon, 2014). The tumor suppressor functions of PTEN have been most thoroughly described in the context of cancers, such as those of the skin, endometrium, prostate and central nervous system (Chalhoub and Baker, 2009). Mounting evidence has documented a role for PTEN in disorders of nervous system function, including ataxia (Backman et al., 2001), seizures (Backman et al., 2001), mental retardation (Varga et al., 2009; McBride et al., 2010), and autism (Varga et al., 2009; Zhou and Parada, 2012). Individuals with germline PTEN mutations are not only prone to cancer, but many of these individuals are also at risk of developing autism with comorbid macrocephaly (Butler et al., 2005; Clipperton-Allen and Page, 2014).

To better understand the cellular and molecular mechanisms by which dysregulated PTEN signaling may predispose individuals to macrocephaly and ASD, multiple groups have genetically engineered PTEN-deficient mice. These studies have shown that mice lacking PTEN in differentiated neurons exhibit macrocephaly, regional hypertrophy, increased soma, dendritic and axonal growth, ectopic axons and dendrites, and increased spine density (Backman et al., 2001; Kwon et al., 2001; Greer and Wynshaw-Boris, 2006; Fraser et al., 2008; Amiri et al., 2012). Additionally, these mice display classic autism-related behavioral abnormalities including impaired social interaction, learning deficits, hypersensitivity to acoustic stimuli, increased seizure susceptibility, and exaggerated anxiety-like behaviors (Kwon et al., 2006). Additionally, loss of PTEN in adult hippocampal stem cell populations was shown to accelerate stem cell proliferation rates and activate PI3K/AKT/mTOR/GSK3B signaling pathways to promote a pro-growth phenotype. Neurons differentiated from these stem cells exhibit dendritic and axonal hypertrophy (Amiri et al., 2012), consistent with PTEN's role as a suppressor of growth in multiple systems. Many of the neuronal hypertrophic phenotypes and behavioral abnormalities associated with loss of PTEN can be rescued by pharmacological inhibition of downstream mTOR signaling with rapamycin, but surprisingly not by loss of S6K1, a downstream effector of ERK1/2 (Kwon et al., 2003; Chalhoub et al., 2006; Zhou et al., 2009). Thus, future research is required to determine the precise mechanisms by which rapamycin-mediated inhibition of mTOR is able to rescue the pro-growth phenotypes observed in PTEN deficient neurons.

### Methyl-CpG-binding protein

Methyl-CpG-binding protein (MeCP2) is a regulator of gene expression which is present in cells throughout the body, but is particularly abundant in the brain. MeCP2 provides an interesting case in which the available evidence indicates that loss of MeCP2 (Rett Syndrome) can produce an “undergrowth” entity, while excess MeCP2 (MeCP2 duplication syndrome) conversely results in a presentation consistent with underlying “overgrowth.” Rett Syndrome (RTT), in 95% of cases, is caused by loss-of-function mutations in the X-linked MeCP2 and is predominately seen in females (around 1/10,000 live births) as these mutations in males lead to perinatal lethality (Amir et al., 1999). Behaviorally, children with Rett syndrome display some core features of autism, including repetitive behaviors and poor communication skills. MeCP2 duplication syndrome, which is caused by duplication of the *MeCP2* gene, presents with intellectual disability, seizures, motor dysfunction, developmental delay and autistic behavior (Ramocki et al., 2010).

Patients with Rett Syndrome appear to develop normally until 6–18 months of life and then exhibit dramatic motor/autonomic deterioration, and deceleration of head growth with microcephaly evident by the second year of life. MeCP2 plays a role in synaptic maturation and pruning in development as well as the maintenance of dendritic arbors in adulthood (Schüle et al., 2008; Matijevic et al., 2009; McGraw et al., 2011; Baj et al., 2014). Characterization of postmortem brain tissue obtained from patients with RTT found a 12–34% reduction in brain weight and volume. There is a decrease in hippocampal spine density, decreased dendritic branching, reduction in neuronal size, and reduced white matter volume. However, there is no obvious degeneration present in RTT brains suggestive of MeCP2's role in regulation of postnatal neuronal growth and not in neurodegeneration (Reiss et al., 1993; Jellinger, 2003). A number of these phenotypes are recapitulated in the male Mecp2-null mice which display normal development until 3–8 weeks of age but begin a sharp decline, exhibiting tremors, difficulty in locomotion and hypotonia. While there are no obvious structural abnormalities present in the MeCP2 null mice, their brains are reduced in size and have a smaller, denser composition of neurons as compared to wild-type controls. While male MeCP2 null mice often die within 6–10 weeks, female heterozygotes remain viable until 4–6 months and at this point begin to exhibit RTT-like symptoms (Guy et al., 2001; Ricceri et al., 2008; Chen et al., 2013). In an additional mouse model of RTT, a truncation of MeCP2 at amino acid 308 (MeCP2308/Y) leads to a less severe progression of neurological phenotypes but also mirrors deficits found in RTT patients. Due to the rare occurrence of MeCP2 duplication syndrome, very little analysis on the morphology of post-mortem brain tissue has been reported; however, some evidence suggests macrocephaly in patients with MeCP2 duplication syndrome (Lugtenberg et al., 2009). The MeCP2 duplication mouse model (2-fold overexpression of human MeCP2) displays an initial enhancement of synaptic plasticity and motor and contextual learning. At around 20 weeks of age, however, these transgenic mice develop seizures and motor deficits and eventually die around 1 year of age (Collins et al., 2004; Luikenhuis et al., 2004). While there are conflicting reports about the effects of MeCP2 overexpression on synaptic density, there is some evidence to suggest that MeCP2 duplication leads to an increased density of excitatory synapses (Zhou et al., 2006; Chao et al., 2007; Chapleau and Pozzo-Miller, 2012). Misexpression of MeCP2 is also associated with other neuropsychiatric disorders ranging from intellectual disability, schizophrenia, Angelman-like syndrome and autism (Chahrour and Zoghbi, 2007).

MeCP2 plays a complex role in regulating the gene expression of multiple growth control pathways, and the cellular outcomes downstream of these genes. Current research, which is detailed in other reviews (Cheng and Qiu, 2014), has shown that MeCP2 can regulate transcription both as a transcriptional repressor, as well as a transcriptional activator through CREB1 recruitment (Chahrour and Zoghbi, 2007). Induced neurons (IN) have been used recently to show that MeCP2-deficiency particularly reduces the levels of activity-dependent neuronal genes which are important for the promotion of growth and plasticity (Li et al., 2013), including BDNF and IGF1 (Castro et al., 2014). *In vitro* MeCP2 has been shown to bind the BDNF promoter region and repress its transcription when the neuron is not active. Following neuronal activity, MeCP2 is phosphorylated at serine 421 and removed from the promoter region, allowing for transcription of BDNF to occur (Chen et al., 2003; Martinowich et al., 2003; Zhou et al., 2006). The story *in vivo*, however, is more complex. In the MeCP2 -/y mice, Chang et al., discovered that overall BDNF levels are reduced, most likely due to reduced cortical activity present in the MeCP2 -/y brain (Dani et al., 2005; Chang et al., 2006). Importantly, the authors demonstrated that increasing expression of BDNF within the forebrain of MeCP2 -/y mice rescues motor function and increases lifespan, suggesting an important functional interaction between this neurotrophic factor and MeCP2. In accordance with this result, loss of MeCP2 has also been reported to depress levels of activated pERK1/2, and activation of the Ras-ERK1/2 pathway by MeCP2 to be required for the promotion of neuronal differentiation by MeCP2 (Sweatt, 2004). MeCP2-deficiency is also reported to lower ribosomal RNA levels and to reduce protein synthesis (Li et al., 2013) consistent with an undergrowth phenotype. Interestingly, recent work provides evidence that MeCP2 interacts with DGCR8, a component of nuclear microRNA (miRNA) processing, and leads to suppression of pri-miRNA processing (Cheng et al., 2014). Effects on miRNA biogenesis could provide an additional regulatory mechanism by which loss of MeCP2 alters not only the quantity, but also the specificity of protein synthesis. Collectively, the cellular and molecular signature of Rett syndrome is consistent with its classification as an undergrowth type ASD, while MeCP2 duplication syndrome is more appropriately categorized as an overgrowth phenotype based on available data.

### Autism susceptibility candidate 2

The autism susceptibility candidate 2 (AUTS2) was initially suspected to be associated with ASD found in a pair of monozygotic twins which had a *de novo* balanced translocation which disrupted the AUTS2 locus (Sultana et al., 2002). It has been reported that a subset of patients harboring deletions within exonic regions of AUTS2 present with a short stature, facial dysmorphism, and microcephaly (Beunders et al., 2013). Given these phenotypes, AUTS2 mutations are thought to lead to predominately “undergrowth” phenotypes. Since the initial identification of the AUTS2 locus, there have been over 30 additional individuals with cognitive disorders ranging from ASD to epilepsy, attention deficit disorder, intellectual disability and developmental delay harboring disruptions in both the coding and noncoding regions of the AUTS2 locus (Kalscheuer et al., 2007; Bakkaloglu et al., 2008; Glessner et al., 2009a; Elia et al., 2010; Pinto et al., 2010; Ben-David et al., 2011). Given that alteration of AUTS2 levels results in a myriad of neuronal deficits, it is not surprising that AUTS2 is highly expressed in developing brain regions important for higher order cognitive functions.

The function of AUTS2 had preliminarily been explored in the model system *Danio rerio* while the recent development of a mouse loss-of-function model has begun to elucidate the function of the *AUTS2* gene product. The function of AUTS2 has been recently addressed in animal models of perturbation in AUTS2. In zebrafish, reductions of AUTS2 by morpholinos knockdown results in microcephaly, reduced mobility and decreases in the number of neurons (Beunders et al., 2013; Oksenberg and Ahituv, 2013). The Auts2 KO mouse model also results in reduction in size and these mice also display developmental delays, motor deficits, and altered communications as assayed by pup ultrasonic vocalizations (USVs) (Gao et al., 2014). This microcephaly phenotype is also observed in mice and human patients with AUTS2 disruptions suggesting a critical role for AUTS2 in neurodevelopment and growth (Beunders et al., 2013).

Previous research has shown AUTS2 to be highly expressed in neurons. The cytoplasmic pool of AUTS2 has been shown to regulate neurite outgrowth and migration through the regulation of the Rho family of GTPases (Hori et al., 2014). Bidirectional alterations of AUTS2 levels in cortical neurons lead to opposing phenotypes in neurite growth. Cortical neurons which have shRNA knockdown of AUTS2 (introduced through in utero electroporation at E15.5 and cultured to DIV 2-6) display decreases in dendrite length and branch number whereas overexpression in primary hippocampal neurons (at DIV 4) leads to increase in neurite elongation (Hori et al., 2014). Nuclear populations of AUTS2 have been shown to play a role in regulation of chromatin dynamics through its interaction with PRC1, highlighting its potential role in regulation of transcription (Gao et al., 2014). One interesting finding from Gao et al. hints at a potential interaction between AUTS2 and signaling pathways involved in growth. The authors find that AUTS2 binds to the promoter regions of factors implicated in growth including TSC2, IGFR, and k-Ras as seen through a ChIP-seq performed in mouse brains. This is suggestive of a mechanism by which disruption of AUTS2 could lead to disruption of neuronal growth and the “undergrowth” phenotype described in human patients and animal models of AUTS2 mutations. While current research has started to address the function of AUTS2 in neuronal growth and development, much work needs to be done to determine the molecular mechanism by which AUTS2 leads to the pathogenesis of autism.

## Copy number variant models of ASD

### 16p11.2 copy number variation

CNV at the human chromosome 16p11.2 locus is among the most common risk variants associated with ASD, accounting for approximately 0.5–1% of all cases (Malhotra and Sebat, 2012). In fact, duplications and deletions in this ~600 kB region of 16p11.2 have been linked to a number of neurodevelopmental and psychiatric conditions, including intellectual disability, schizophrenia, epilepsy, bipolar disorder, and obesity (Weiss et al., 2008; McCarthy et al., 2009; Shinawi et al., 2010; Walters et al., 2010; Zufferey et al., 2012). Several reports indicate that reciprocal phenotypes occur as a result of deletion or duplication of the approximately 27 protein-coding genes found in the affected region. Therefore, 16p11.2 CNVs present a unique model to explore the effects of altered dosage of genes within this region on underlying growth pathway correlates.

In humans, there is accumulating evidence supporting the presence of pro-growth phenotypes in deletion carriers and anti-growth features in duplication carriers. For instance, 16p11.2 microdeletion is linked to macrocephaly, whereas duplication of this locus has been associated with microcephaly (Shinawi et al., 2010). A recent study comparing global brain differences between child carriers of 16p11.2 deletion (8.1 ± 3 years) and duplication (7.8 ± 5 years) identified a strong dose-dependent influence of CNV on brain volume. In deletion carriers, global measures of gray and white matter volume, along with volumes of certain subcortical structures such as the thalamus, were found to be significantly elevated as compared to typically developing controls. In contrast, these measures were altered in the opposite direction in the case of duplication carriers (Qureshi et al., 2014).

CNVs at 16p11.2 also confer highly penetrant and opposing effects on body mass index (BMI). Parallel to the effects observed with head size, deletion of this region often results in early-onset obesity, while duplication is associated with significantly reduced postnatal body weight and BMI (Jacquemont et al., 2011). Importantly, a study that analyzed the co-occurrence of head size and BMI phenotypes observed a higher incidence of the 16p11.2 deletion among cohorts ascertained for both developmental delay and obesity (2.9%), as opposed to cohorts assessed for either outcome alone (0.6 and 0.4%, respectively) (Walters et al., 2010). A potential avenue for future investigation in mouse model systems is to test the involvement of common molecular pathways in both neuronal and somatic growth defects in 16p11.2 CNVs.

In order to systematically assess the effects of this CNV on development and behavior, two independent groups, Horev et al. and Portmann et al., utilized chromosome engineering approaches to develop mutant mouse models harboring deletion (*df/*+) and/or duplication (*dp/*+) of the conserved syntenic region on chromosome 7 (Horev et al., 2011; Portmann et al., 2014). Characterization of adult *df/*+ and *dp/*+ mutants has revealed dose-dependent effects on expression of most genes within the engineered interval, as well as opposing phenotypes in certain neuroanatomical measures, including mild reciprocal volumetric changes across multiple brain regions in adulthood (Horev et al., 2011).

However, currently available mouse models of 16p11.2 CNVs may not accurately recapitulate head and body size phenotypes observed in human carriers. Despite expected changes in expression of genes in the deleted interval, *df/*+mutants display decreases in postnatal body weight (Portmann et al., 2014; Pucilowska et al., 2015) and mild reductions in overall brain volume that persist into adulthood (Horev et al., 2011; Portmann et al., 2014; Pucilowska et al., 2015). Therefore, more detailed phenotypic characterization of 16p11.2 CNV mouse models is required in order to pinpoint the cause for this discrepancy.

The gene, *MAPK3*, which encodes ERK1 is located within the 16p11.2 region and has garnered interest with respect to molecular phenotypes associated with this CNV. However, there have been conflicting reports on the effect of 16p11.2 deletion, and consequent ERK1 deficiency, on overall ERK1/2 activity. When ERK1/2 activity is measured as a ratio of phosphorylated to total ERK1/2 levels, studies have reported contradicting findings. One group observed decreases in ERK1/2 activity, which were correlated with decreased overall protein synthesis (Tian et al., 2015). On the other hand, Pucilowska et al., found a paradoxical upregulation of ERK1/2 activation (Pucilowska et al., 2015). Inconsistencies in reports may necessitate the use of ERK1/2 reporters to accurately assay ERK1/2 catalytic function in mouse models of 16p11.2 CNVs. Elucidating ERK1/2 pathway readouts in 16p11.2 CNVs is further complicated by the fact that a gene known to function as a negative regulator of ERK1/2, known as major vault protein (MVP), is also found within the 16p11.2 locus (Liang et al., 2010). Therefore, further studies need to be conducted in order to ascertain the impact of 16p11.2 CNVs on readouts of ERK1/2 activity.

### 22q11.2 copy number variation

Several reports have described the co-occurrence of autism in subjects with chromosome 22 abnormalities including trisomy 22, translocation 20/22, 22q11.2 deletion, 22q11.2 duplication, ring chromosome 22, and 22q13.3 deletion (Mukaddes and Herguner, 2007). In 22q11.2 microdeletion (22q11.2DS; velocardiofacial syndrome, Digeorge syndrome), a syndrome characterized by either a 1.5 or 3 megabasepair deletion of 22q11, roughly one third of individuals develop a form of schizophrenia and a smaller fraction (~10–20%) display developmental delays and learning disabilities characteristic of autism. In addition to microdeletion at the 22q11.2 locus, genome-wide assays for copy number variants have also identified significant enrichment of 22q11.2 duplication in unrelated ASD patients (Marshall et al., 2008; Glessner et al., 2009b).

Although neuroimaging studies of patients with 22q11.2DS report a wide range of abnormalities in both brain structure and function, which are consistent with neuronal undergrowth phenotypes, the literature remains relatively inconsistent regarding differences in specific neuronal regions or structures. Thus, direct correlations between these structural changes and behavior are unable to be made at this time. These different conclusions may be due to the use of different protocols for measuring or normalizing volumes in studies that should be replicated with larger cohorts (Karayiorgou et al., 2010). However, in a meta-review on 22q11.2DS, when Karayiorgou et al. examined those findings that have been replicated by at least two independent research groups in at least two separate and independent studies, they observed a pattern of neuroanatomical abnormalities associated with undergrowth phenotypes in patients with 22q11.2DS, both with and without psychosis (Karayiorgou et al., 2010). These changes include larger ventricles, reduced brain size, and volumetric reductions of parietal lobes, hippocampus, cerebellum, reduced cortical thickness, and lateral cortical thinning (Xu et al., 2010; Drew et al., 2011a). Recent data modeling the 22q11.2 deletion in rodents has revealed that a significant number of these anatomical findings from 22q11.2 patients, including impaired circuitry and reduced regional volumes of brain structures that are critical to autism, are reliably recapitulated in the brains of mice modeling this human disorder (Sigurdsson et al., 2010; Drew et al., 2011a; Ellegood et al., 2014).

The 22q11.2DS mouse displays neuronal undergrowth, dysregulated synaptic plasticity, and impaired neuronal circuit formation, which are associated with abnormal behavioral outcomes (Karayiorgou et al., 2010; Drew et al., 2011b; Ellegood et al., 2014); however, the precise genetic mechanisms underlying this phenotype remain unknown. This region of human chromosome 22q11 contains 16 protein coding genes, many of which have been shown to regulate cellular growth and proliferation, such as *TBX1, RTN4R*, and *ZDHHC8* (Yagi et al., 2003; Mukai et al., 2008; Borrie et al., 2012); however, for the purposes of this review, we will focus on another gene found within this locus, *DGCR8*, since deletion of *DGCR8* in mouse models has revealed neuronal undergrowth that is able to be rescued by enhanced trophic signaling.

DGCR8 positively regulates the maturation of miRNAs, a recently discovered class of small, highly evolutionarily conserved regulatory RNAs, which inhibit the translation of near-complementary target mRNAs (Han et al., 2006). Individuals with hemizygous expression of DGCR8, as found in 22q11.2 deletion patients, would thus have impaired miRNA biogenesis and reduced inhibition of target protein synthesis, while those with 22q11.2 microduplication will have increased DGCR8 gene dosage (Xu et al., 2010). In mice with DGCR8 haploinsufficiency, there is reduced basal dendritic complexity (fewer branch points, less intersections, and decreased overall dendritic length), decreased hippocampal neurogenesis, and significant cognitive and behavioral deficits, consistent with the neuronal undergrowth phenotype present in individuals with 22q11.2 microdeletion (Schofield et al., 2011; Ouchi et al., 2013). Although it remains unknown as to whether or not these neuronal growth deficits and behavioral abnormalities are a direct consequence of reduced miRNA levels and enhanced translation of these miRNA target genes, subsequent experiments revealed that many of these deficits can indeed be rescued by exogenous application of insulin-like growth factor 2 (Igf2), a gene that is reduced in DGCR8-deficient animals by an unknown mechanism (Ouchi et al., 2013). It should also be noted that while expression of a phosphomimetic DGCR8 (which mimics phosphorylation by kinases such as MAPK/ERK) induces a pro-growth miRNA signature in a heterologous cell line (Herbert et al., 2013), it remains unknown whether or not the phosphorylation status of DGCR8 could serve as a diagnostic biomarker for cases of autism characterized by aberrant growth.

## Conclusions

### Relevance for future clinical research and trials

A compelling avenue of investigation is to design therapeutics that effectively target pathways controlling neuronal growth in ASD. Recent studies have allowed us to make significant progress in understanding the nature of neuronal growth defects in ASD. It remains a significant question in the field as to whether growth defects are causal to the development and pathogenesis of ASD. There are two lines of evidence that suggest that this may be the case (i) growth abnormalities emerge early in the pathogenesis of the disease, and (ii) manipulations and interventions that target growth-related pathways not only ameliorate growth defects but also lead to improvements in behavior.

For therapies to yield maximal success, the initial identification and design should consider whether the form of ASD being treated could be generally classified as overgrowth or undergrowth. This sub-classification of growth phenotypes in ASD is critical as the same drug is unlikely to be effective under both circumstances. Aside from obvious growth defects such as macrocephaly and microcephaly, more subtle growth phenotypes might prove to be difficult to diagnose early in development. Ultimately, development of a biomarker that serves as a readout for growth status in ASD would be necessary in order to facilitate early detection and proper intervention. Possible development of biomarkers could include components of growth pathways highlighted in this review, such as ERK1/2, mTOR, and neurotrophic factors.

### Relevance for future research efforts

Proper growth of functional neural circuits requires highly orchestrated control of intracellular signaling cascades and gene expression. These changes in neuronal growth depend upon the specific composition of gene regulatory networks present not only within each cell type, but within subcellular regions of a given neuron that give rise to the various neuronal processes, such as axons, dendrites, and spines, structures that are often dysregulated in forms of autism characterized by over- or undergrowth. Although CRE-driver lines have been highly informative for our understanding of cell type specific changes in behavior, gene expression, patterns of neuronal growth, and connectivity across various brain regions between wild-type mice and disease models, development of CRE-drivers requires a priori knowledge of the specific gene expression patterns unique to each cell type. Even within cells identified by such a CRE-driver system, there exists considerable heterogeneity in terms of gene expression. Each of the human brain's billions of neurons is unique not only in terms of its activity signature but also within the complex three-dimensional spatial environment of the brain that permits unique connectivity for each neuron. For these reasons, we predict that future single cell analyses of gene expression across ensembles of cells from wild-type controls and disease models will transform our understanding of these highly complex cells and gain better mechanistic insights into the signaling pathways that underlie over- or under-growth phenotypes in ASD.

Many large-scale ASD studies have now been conducted, which have included mixed patient populations, with variations in multiple critical factors including age, diagnosis, and symptom severity. However, it has remained difficult to extract meaningful conclusions from these studies, in part, since molecular changes are likely to be different among forms of ASD with differing underlying etiologies. Identifying common molecular pathways that are dysregulated among various genetic causes of ASD is an important step in effectively stratifying ASD into endophenotypes from which therapeutic responses can be more readily anticipated. Shrinking the heterogeneity of the disorder would also make the study of ASD more tractable, allowing investigations in monogenic mouse models to be more appropriately applied to other genetic forms of ASD. Utilizing overgrowth and undergrowth phenotypes (Figure 1) as a basis for this stratification is useful since these phenotypes are present across multiple genetic causes of ASD and occur early in the development of disease.

### Conflict of interest statement

The authors declare that the research was conducted in the absence of any commercial or financial relationships that could be construed as a potential conflict of interest.

## References

[B1] Abrahams B. S. Geschwind D. H. (2008). Advances in autism genetics: on the threshold of a new neurobiology. Nat. Rev. Genet. 9, 341–355. 10.1038/nrg2346 18414403 PMC2756414

[B2] Amir R. E. Van den Veyver I. B. Wan M. Tran C. Q. Francke U. Zoghbi H. Y. (1999). Rett syndrome is caused by mutations in X-linked MECP2, encoding methyl-CpG-binding protein 2. Nat. Genet. 23, 185–188. 10.1038/13810 10508514

[B3] Amiri A. Cho W. Zhou J. Birnbaum S. G. Sinton C. M. McKay R. M.. (2012). Pten deletion in adult hippocampal neural stem/progenitor cells causes cellular abnormalities and alters neurogenesis. J. Neurosci. 32, 5880–5890. 10.1523/JNEUROSCI.5462-11.2012 22539849 PMC6703621

[B4] Anderson J. S. (2014). Cortical underconnectivity hypothesis in autism: evidence from functional connectivity MRI, in Comprehensive Guide to Autism, eds Patel V. B. Preedy V. R. Martin C. R. (New York, NY: Springer Science+Business Media), 1457–1471. 10.1007/978-1-4614-4788-7_81

[B5] Anderson J. S. Druzgal T. J. Froehlich A. Dubray M. B. Lange N. Alexander A. L.. (2011). Decreased interhemispheric functional connectivity in autism. Cereb. Cortex 21, 1134–1146. 10.1093/cercor/bhq190 20943668 PMC3077433

[B6] Antion M. D. Hou L. Wong H. Hoeffer C. A. Klann E. (2008). mGluR-dependent long-term depression is associated with increased phosphorylation of S6 and synthesis of elongation factor 1A but remains expressed in S6K-deficient mice. Mol. Cell. Biol. 28, 2996–3007. 10.1128/MCB.00201-08 18316404 PMC2293080

[B7] Assaf M. Jagannathan K. Calhoun V. D. Miller L. Stevens M. C. Sahl R.. (2010). Abnormal functional connectivity of default mode sub-networks in autism spectrum disorder patients. Neuroimage 53, 247–256. 10.1016/j.neuroimage.2010.05.067 20621638 PMC3058935

[B8] Auerbach B. D. Osterweil E. K. Bear M. F. (2011). Mutations causing syndromic autism define an axis of synaptic pathophysiology. Nature 480, 63–68. 10.1038/nature10658 22113615 PMC3228874

[B9] Backman S. A. Stambolic V. Suzuki A. Haight J. Elia A. Pretorius J.. (2001). Deletion of Pten in mouse brain causes seizures, ataxia and defects in soma size resembling Lhermitte-Duclos disease. Nat. Genet. 29, 396–403. 10.1038/ng782 11726926

[B10] Baj G. Patrizio A. Montalbano A. Sciancalepore M. Tongiorgi E. (2014). Developmental and maintenance defects in Rett syndrome neurons identified by a new mouse staging system *in vitro*. Front. Cell. Neurosci. 8:18. 10.3389/fncel.2014.00018 24550777 PMC3914021

[B11] Bakkaloglu B. O'Roak B. J. Louvi A. Gupta A. R. Abelson J. F. Morgan T. M.. (2008). Molecular cytogenetic analysis and resequencing of contactin associated protein-like 2 in autism spectrum disorders. Am. J. Hum. Genet. 82, 165–173. 10.1016/j.ajhg.2007.09.017 18179895 PMC2253974

[B12] Banko J. L. Hou L. Poulin F. Sonenberg N. Klann E. (2006). Regulation of eukaryotic initiation factor 4E by converging signaling pathways during metabotropic glutamate receptor-dependent long-term depression. J. Neurosci. 26, 2167–2173. 10.1523/JNEUROSCI.5196-05.2006 16495443 PMC6674817

[B13] Banko J. L. Poulin F. Hou L. DeMaria C. T. Sonenberg N. Klann E. (2005). The translation repressor 4E-BP2 is critical for eIF4F complex formation, synaptic plasticity, and memory in the hippocampus. J. Neurosci. 25, 9581–9590. 10.1523/JNEUROSCI.2423-05.2005 16237163 PMC6725736

[B14] Bartholomeusz H. H. Courchesne E. Karns C. M. (2002). Relationship between head circumference and brain volume in healthy normal toddlers, children, and adults. Neuropediatrics 33, 239–241. 10.1055/s-2002-36735 12536365

[B15] Bateup H. S. Johnson C. A. Denefrio C. L. Saulnier J. L. Kornacker K. Sabatini B. L. (2013). Excitatory/Inhibitory synaptic imbalance leads to hippocampal hyperexcitability in mouse models of tuberous sclerosis. Neuron 78, 510–522. 10.1016/j.neuron.2013.03.017 23664616 PMC3690324

[B16] Ben-David E. Granot-Hershkovitz E. Monderer-Rothkoff G. Lerer E. Levi S. Yaari M.. (2011). Identification of a functional rare variant in autism using genome-wide screen for monoallelic expression. Hum. Mol. Genet. 20, 3632–3641. 10.1093/hmg/ddr283 21680558

[B17] Bernier R. Golzio C. Xiong B. Stessman H. A. Coe B. P. Penn O.. (2014). Disruptive CHD8 mutations define a subtype of autism early in development. Cell 158, 263–276. 10.1016/j.cell.2014.06.017 24998929 PMC4136921

[B18] Beunders G. Voorhoeve E. Golzio C. Pardo L. M. Rosenfeld J. A. Talkowski M. E.. (2013). Exonic deletions in AUTS2 cause a syndromic form of intellectual disability and suggest a critical role for the C terminus. Am. J. Hum. Genet. 92, 210–220. 10.1016/j.ajhg.2012.12.011 23332918 PMC3567268

[B19] Bhakar A. L. Dölen G. Bear M. F. (2012). The pathophysiology of fragile X (and what it teaches us about synapses). Annu. Rev. Neurosci. 35, 417–443. 10.1146/annurev-neuro-060909-153138 22483044 PMC4327822

[B20] Bhattacharya A. Kaphzan H. Alvarez-Dieppa A. C. Murphy J. P. Pierre P. Klann E. (2012). Genetic removal of p70 S6 kinase 1 corrects molecular, synaptic, and behavioral phenotypes in fragile X syndrome mice. Neuron 76, 325–337. 10.1016/j.neuron.2012.07.022 23083736 PMC3479445

[B21] Bilousova T. V. Dansie L. Ngo M. Aye J. Charles J. R. Ethell D. W.. (2009). Minocycline promotes dendritic spine maturation and improves behavioural performance in the fragile X mouse model. J. Med. Genet. 46, 94–102. 10.1136/jmg.2008.061796 18835858

[B22] Borrie S. C. Baeumer B. E. Bandtlow C. E. (2012). The Nogo-66 receptor family in the intact and diseased CNS. Cell Tissue Res. 349, 105–117. 10.1007/s00441-012-1332-9 22311207

[B23] Bronicki L. M. Redin C. Drunat S. Piton A. Lyons M. Passemard S.. (2015). Ten new cases further delineate the syndromic intellectual disability phenotype caused by mutations in DYRK1A. Eur. J. Hum. Genet.. 10.1038/ejhg.2015.29. [Epub ahead of print]. 25920557 PMC4613470

[B24] Butler M. G. Dasouki M. J. Zhou X.-P. Talebizadeh Z. Brown M. Takahashi T. N.. (2005). Subset of individuals with autism spectrum disorders and extreme macrocephaly associated with germline PTEN tumour suppressor gene mutations. J. Med. Genet. 42, 318–321. 10.1136/jmg.2004.024646 15805158 PMC1736032

[B25] Byts N. Samoylenko A. Woldt H. Ehrenreich H. Sirén A. L. (2006). Cell type specific signalling by hematopoietic growth factors in neural cells. Neurochem. Res. 31, 1219–1230. 10.1007/s11064-006-9149-0 17021950

[B26] Çaku A. Pellerin D. Bouvier P. Riou E. Corbin F. (2014). Effect of lovastatin on behavior in children and adults with fragile X syndrome: an open-label study. Am. J. Med. Genet. A, 164, 2834–2842. 10.1002/ajmg.a.36750 25258112

[B27] Callan M. A. Cabernard C. Heck J. Luois S. Doe C. Q. Zarnescu D. C. (2010). Fragile X protein controls neural stem cell proliferation in the Drosophila brain. Hum. Mol. Genet. 19, 3068–3079. 10.1093/hmg/ddq213 20504994 PMC2901145

[B28] Castrén M. Castrén E. (2014). BDNF in fragile X syndrome. Neuropharmacology 76 Pt C, 729–736. 10.1016/j.neuropharm.2013.05.018 23727436

[B29] Castrén M. Tervonen T. Kärkkäinen V. Heinonen S. Castrén E. Larsson K.. (2005). Altered differentiation of neural stem cells in fragile X syndrome. Proc. Natl. Acad. Sci. U.S.A. 102, 17834–19839. 10.1073/pnas.0508995102 16314562 PMC1308923

[B30] Castro J. Garcia R. I. Kwok S. Banerjee A. Petravicz J. Woodson J.. (2014). Functional recovery with recombinant human IGF1 treatment in a mouse model of Rett Syndrome. Proc. Natl. Acad. Sci. U.S.A. 111, 9941–9946. 10.1073/pnas.1311685111 24958891 PMC4103342

[B31] Chahrour M. Zoghbi H. Y. (2007). The story of rett syndrome: from clinic to neurobiology. Neuron 56, 422–437. 10.1016/j.neuron.2007.10.001 17988628

[B32] Chalhoub N. Baker S. J. (2009). PTEN and the PI3-kinase pathway in cancer. Annu. Rev. Pathol. 4, 127–150. 10.1146/annurev.pathol.4.110807.092311 18767981 PMC2710138

[B33] Chalhoub N. Kozma S. C. Baker S. J. (2006). S6k1 is not required for Pten-deficient neuronal hypertrophy. Brain Res. 1100, 32–41. 10.1016/j.brainres.2006.05.013 16777079

[B34] Chang Q. Khare G. Dani V. Nelson S. Jaenisch R. (2006). The disease progression of Mecp2 mutant mice is affected by the level of BDNF expression. Neuron 49, 341–348. 10.1016/j.neuron.2005.12.027 16446138

[B35] Chao H. T. Zoghbi H. Y. Rosenmund C. (2007). MeCP2 controls excitatory synaptic strength by regulating glutamatergic synapse number. Neuron 56, 58–65. 10.1016/j.neuron.2007.08.018 17920015 PMC2198899

[B36] Chao M. V. Rajagopal R. Lee F. S. (2006). Neurotrophin signalling in health and disease. Clin. Scie. (Lond.) 110, 167–173. 10.1042/CS20050163 16411893

[B37] Chapleau C. A. Pozzo-Miller L. (2012). Divergent roles of p75 NTR and Trk receptors in BDNF's effects on dendritic spine density and morphology. Neural Plast. 2012:578057. 10.1155/2012/578057 22548193 PMC3323862

[B38] Chen W. G. Chang Q. Lin Y. Meissner A. West A. E. Griffith E. C.. (2003). Derepression of BDNF transcription involves calcium-dependent phosphorylation of MeCP2. Science 302, 885–889. 10.1126/science.1086446 14593183

[B39] Chen Y. H. Liao D. L. Lai C. H. Chen C. H. (2013). Genetic analysis of AUTS2 as a susceptibility gene of heroin dependence. Drug Alcohol Depend. 128, 238–242. 10.1016/j.drugalcdep.2012.08.029 22995765

[B40] Cheng T.-L. Qiu Z. (2014). MeCP2: multifaceted roles in gene regulation and neural development. Neurosci. Bull. 30, 601–609. 10.1007/s12264-014-1452-6 25082535 PMC5562630

[B41] Cheng T. L. Wang Z. Liao Q. Zhu Y. Zhou W. H. Xu W.. (2014). MeCP2 Suppresses nuclear microRNA processing and dendritic growth by regulating the DGCR8/Drosha complex. Dev. Cell 28, 547–560. 10.1016/j.devcel.2014.01.032 24636259

[B42] Chévere-Torres I. Kaphzan H. Bhattacharya A. Kang A. Maki J. M. Gambello M. J.. (2012). Metabotropic glutamate receptor-dependent long-term depression is impaired due to elevated ERK signaling in the DeltaRG mouse model of tuberous sclerosis complex. Neurobiol. Dis. 45, 1101–1110. 10.1016/j.nbd.2011.12.028 22198573 PMC3276695

[B43] Choi Y.-J. Di Nardo A. Kramvis I. Meikle L. Kwiatkowski D. J. Sahin M.. (2008). Tuberous sclerosis complex proteins control axon formation. Genes Dev. 22, 2485–2495. 10.1101/gad.1685008 18794346 PMC2546692

[B44] Chudley A. Hagerman R. (1987). Fragile X syndrome. J. Petriatrics 110, 821–831. 10.1016/s0022-3476(87)80392-x 3295158

[B45] Clipperton-Allen A. E. Page D. T. (2014). Pten haploinsufficient mice show broad brain overgrowth but selective impairments in autism-relevant behavioral tests. Hum. Mol. Genet. 23, 3490–3505. 10.1093/hmg/ddu057 24497577

[B46] Cloëtta D. Thomanetz V. Baranek C. Lustenberger R. M. Lin S. Oliveri F.. (2013). Inactivation of mTORC1 in the developing brain causes microcephaly and affects gliogenesis. J. Neurosci. 33, 7799–7810. 10.1523/JNEUROSCI.3294-12.2013 23637172 PMC6618947

[B47] Collins A. L. Levenson J. M. Vilaythong A. P. Richman R. Armstrong D. L. Noebels J. L.. (2004). Mild overexpression of MeCP2 causes a progressive neurological disorder in mice. Hum. Mol. Genet. 13, 2679–2689. 10.1093/hmg/ddh282 15351775

[B48] Courchesne E. Carper R. Akshoomoff N. (2003). Evidence of brain overgrowth in the first year of life in autism. J. Am. Med. Assoc. 290, 337–344. 10.1001/jama.290.3.337 12865374

[B49] Courchesne E. Pierce K. Schumann C. M. Redcay E. Buckwalter J. A. Kennedy D. P.. (2007). Mapping early brain development in autism. Neuron, 56, 399–413. 10.1016/j.neuron.2007.10.016 17964254

[B50] Cruz-Martín A. Crespo M. Portera-Cailliau C. (2010). Delayed stabilization of dendritic spines in fragile X mice. J. Neurosci. 30, 7793–7803. 10.1523/JNEUROSCI.0577-10.2010 20534828 PMC2903441

[B51] Curatolo P. Bombardieri R. Jozwiak S. (2008). Tuberous sclerosis. Lancet 372, 657–668. 10.1016/S0140-6736(08)61279-9 18722871

[B52] Dani V. S. Chang Q. Maffei A. Turrigiano G. G. Jaenisch R. Nelson S. B. (2005). Reduced cortical activity due to a shift in the balance between excitation and inhibition in a mouse model of Rett syndrome. Proc. Natl. Acad. Sci. U.S.A. 102, 12560–12565. 10.1073/pnas.0506071102 16116096 PMC1194957

[B53] Darnell J. C. Van Driesche S. J. Zhang C. Hung K. Y. S. Mele A. Fraser C. E.. (2011). FMRP stalls ribosomal translocation on mRNAs linked to synaptic function and autism. Cell 146, 247–261. 10.1016/j.cell.2011.06.013 21784246 PMC3232425

[B54] Dawbarn D. Allen S. J. (2003). Neurotrophins and neurodegeneration. Neuropathol. Appl. Neurobiol. 29, 211–230. 10.1046/j.1365-2990.2003.00487.x 12787319

[B55] De Rubeis S. He X. Goldberg A. P. Poultney C. S. Samocha K. Ercument Cicek A.. (2014). Synaptic, transcriptional and chromatin genes disrupted in autism. Nature 515, 209–215. 10.1038/nature13772 25363760 PMC4402723

[B56] Dölen G. Osterweil E. Rao B. S. Smith G. B. Auerbach B. D. Chattarji S.. (2007). Correction of fragile X syndrome in mice. Neuron, 56, 955–962. 10.1016/j.neuron.2007.12.001 18093519 PMC2199268

[B57] Drew L. J. Crabtree G. W. Markx S. Stark K. L. Chaverneff F. Xu B.. (2011a). The 22q11.2 microdeletion: Fifteen years of insights into the genetic and neural complexity of psychiatric disorders. Int. J. Dev. Neurosci. 29, 259–281. 10.1016/j.ijdevneu.2010.09.007 20920576 PMC3074020

[B58] Drew L. J. Stark K. L. Fénelon K. Karayiorgou M. Macdermott A. B. Gogos J. A. (2011b). Evidence for altered hippocampal function in a mouse model of the human 22q11.2 microdeletion. Mol. Cell. Neurosci. 47, 293–305. 10.1016/j.mcn.2011.05.008 21635953 PMC3539311

[B59] Dziembowska M. Pretto D. I. Janusz A. Kaczmarek L. Leigh M. J. Gabriel N.. (2013). High MMP-9 activity levels in fragile X syndrome are lowered by minocycline. Am. J. Med. Genet. A, 161, 1897–1903. 10.1002/ajmg.a.36023 23824974

[B60] Eapen V. Clarke R. A. (2014). Autism spectrum disorders: from genotypes to phenotypes. Front. Hum. Neurosci. 8:914. 10.3389/fnhum.2014.00914 25429265 PMC4228832

[B61] Ehninger D. Han S. Shilyansky C. Zhou Y. Li W. Kwiatkowski D. J.. (2008). Reversal of learning deficits in a Tsc2+/- mouse model of tuberous sclerosis. Nat. Med. 14, 843–848. 10.1038/nm1788 18568033 PMC2664098

[B62] Elia J. Gai X. Xie H. M. Perin J. C. Geiger E. Glessner J. T.. (2010). Rare structural variants found in attention-deficit hyperactivity disorder are preferentially associated with neurodevelopmental genes. Mol. Psychiatry 15, 637–646. 10.1038/mp.2009.57 19546859 PMC2877197

[B63] Ellegood J. Anagnostou E. Babineau B. A. Crawley J. N. Lin L. Genestine M.. (2015). Clustering autism: using neuroanatomical differences in 26 mouse models to gain insight into the heterogeneity. Mol. Psychiatry 20, 118–125. 10.1038/mp.2014.98 25199916 PMC4426202

[B64] Ellegood J. Markx S. Lerch J. P. Steadman P. E. Genç C. Provenzano F.. (2014). Neuroanatomical phenotypes in a mouse model of the 22q11.2 microdeletion. Mol. Psychiatry 19, 99–107. 10.1038/mp.2013.112 23999526 PMC3872255

[B65] Faridar A. Jones-davis D. Rider E. Li J. Gobius I. Morcom L.. (2014). *Mapk*/Erk activation in an animal model of social deficits shows a possible link to Autism. Mol. Autism 5:57. 10.1186/2040-2392-5-57 25874073 PMC4396809

[B66] Ferrara N. Gerber H. P. LeCouter J. (2003). The biology of VEGF and its receptors. Nat. Med. 9, 669–676. 10.1038/nm0603-669 12778165

[B67] Fombonne E. Rogé B. Claverie J. Courty S. Frémolle J. (1999). Microcephaly and macrocephaly in autism. J. Autism Dev. Disord. 29, 113–119. 10.1023/A:1023036509476 10382131

[B68] Fraser M. M. Bayazitov I. T. Zakharenko S. S. Baker S. J. (2008). Phosphatase and tensin homolog, deleted on chromosome 10 deficiency in brain causes defects in synaptic structure, transmission and plasticity, and myelination abnormalities. Neuroscience 151, 476–488. 10.1016/j.neuroscience.2007.10.048 18082964 PMC2278004

[B69] Gadow K. D. Roohi J. Devincent C. J. Kirsch S. Hatchwell E. (2009). Association of COMT (Val158Met) and BDNF (Val66Met) gene polymorphisms with anxiety, ADHD and tics in children with autism spectrum disorder. J. Autism Dev. Disord. 39, 1542–1551. 10.1007/s10803-009-0794-4 19582565 PMC4348067

[B70] Gao Z. Lee P. Stafford J. M. von Schimmelmann M. Schaefer A. Reinberg D. (2014). An AUTS2–Polycomb complex activates gene expression in the CNS. Nature 516, 349–354. 10.1038/nature13921 25519132 PMC4323097

[B71] Gasparini L. Xu H. (2003). Potential roles of insulin and IGF-1 in Alzheimer's disease. Trends Neurosci. 26, 404–406. 10.1016/S0166-2236(03)00163-2 12900169

[B72] Gibson J. R. Bartley A. F. Hays S. A. Huber K. M. (2008). Imbalance of neocortical excitation and inhibition and altered UP states reflect network hyperexcitability in the mouse model of fragile X syndrome. J. Neurophysiol. 100, 2615–2626. 10.1152/jn.90752.2008 18784272 PMC2585391

[B73] Gilman S. R. Iossifov I. Levy D. Ronemus M. Wigler M. Vitkup D. (2011). Rare *De novo* variants associated with autism implicate a large functional network of genes involved in formation and function of synapses. Neuron 70, 898–907. 10.1016/j.neuron.2011.05.021 21658583 PMC3607702

[B74] Gkogkas C. G. Khoutorsky A. Cao R. Jafarnejad S. M. Prager-Khoutorsky M. Giannakas N.. (2014). Pharmacogenetic inhibition of eIF4E-Dependent Mmp9 mRNA translation reverses Fragile X syndrome-like phenotypes. Cell Rep. 9, 1742–1755. 10.1016/j.celrep.2014.10.064 25466251 PMC4294557

[B75] Gkogkas C. G. Khoutorsky A. Ran I. Rampakakis E. Nevarko T. Weatherill D. B.. (2013). Autism-related deficits via dysregulated eIF4E-dependent translational control. Nature 493, 371–377. 10.1038/nature11628 23172145 PMC4133997

[B76] Glessner J. T. Wang K. Cai G. Korvatska O. Kim C. E. Wood S.. (2009a). Autism genome-wide copy number variation reveals ubiquitin and neuronal genes. Nature 459, 569–573. 10.1038/nature07953 19404257 PMC2925224

[B77] Glessner J. T. Wang K. Cai G. Korvatska O. Kim C. E. Wood S.. (2009b). Autism genome-wide copy number variation reveals ubiquitin and neuronal genes. Nature 459, 569–573. 10.1038/nature07953 19404257 PMC2925224

[B78] Goldin M. Segal M. (2003). Protein kinase C and ERK involvement in dendritic spine plasticity in cultured rodent hippocampal neurons. Eur. J. Neurosci. 17, 2529–2539. 10.1046/j.1460-9568.2003.02694.x 12823460

[B79] Gonçalves J. T. Anstey J. E. Golshani P. Portera-Cailliau C. (2013). Circuit level defects in the developing neocortex of Fragile X mice. Nat. Neurosci. 16, 903–909. 10.1038/nn.3415 23727819 PMC3695061

[B80] Goorden S. M. I. Van Woerden G. M. Van Der Weerd L. Cheadle J. P. Elgersma Y. (2007). Cognitive deficits in Tsc1+/- mice in the absence of cerebral lesions and seizures. Ann. Neurol. 62, 648–655. 10.1002/ana.21317 18067135

[B81] Govindarajan B. Mizesko M. C. Miller M. S. Onda H. Nunnelly M. Casper K. (2003). Tuberous sclerosis-associated neoplasms express activated p42/44 mitogen-activated protein (MAP) kinase, and inhibition of MAP kinase signaling results in decreased *in vivo* tumor growth. Clin. Cancer Res. 9, 3469–3475. Available online at: clincancerres.aacrjournals.org/content/9/9/3469.long 12960139

[B82] Greer J. M. Wynshaw-Boris A. (2006). Pten and the brain: sizing up social interaction. Neuron 50, 343–345. 10.1016/j.neuron.2006.04.021 16675386

[B83] Gross C. Raj N. Molinaro G. Allen A. G. Whyte A. J. Gibson J. R.. (2015). Selective role of the catalytic PI3K subunit p110β in impaired higher order cognition in Fragile X syndrome. Cell Rep. 11, 681–688. 10.1016/j.celrep.2015.03.065 25921527 PMC4426038

[B84] Guertin D. A. Stevens D. M. Thoreen C. C. Burds A. A. Kalaany N. Y. Moffat J.. (2006). Ablation in Mice of the mTORC components raptor, rictor, or mLST8 reveals that mTORC2 is required for signaling to Akt-FOXO and PKC, but not S6K1. Dev. Cell 11, 859–871. 10.1016/j.devcel.2006.10.007 17141160

[B85] Guy J. Hendrich B. Holmes M. Martin J. E. Bird A. (2001). A mouse Mecp2-null mutation causes neurological symptoms that mimic Rett syndrome. Nat. Genet. 27, 322–326. 10.1038/85899 11242117

[B86] Han J. Lee Y. Yeom K. H. Nam J. W. Heo I. Rhee J. K.. (2006). Molecular basis for the recognition of primary microRNAs by the Drosha-DGCR8 complex. Cell 125, 887–901. 10.1016/j.cell.2006.03.043 16751099

[B87] Harlow E. G. Till S. M. Russell T. A. Wijetunge L. S. Kind P. Contractor A. (2010). Critical period plasticity is disrupted in the barrel cortex of Fmr1 knockout mice. Neuron 65, 385–398. 10.1016/j.neuron.2010.01.024 20159451 PMC2825250

[B88] Hay N. (2005). The Akt-mTOR tango and its relevance to cancer. Cancer Cell 8, 179–183. 10.1016/j.ccr.2005.08.008 16169463

[B89] He C. X. Portera-Cailliau C. (2013). The trouble with spines in fragile X syndrome: density, maturity and plasticity. Neuroscience 251, 120–128. 10.1016/j.neuroscience.2012.03.049 22522472 PMC3422423

[B90] He Q. Nomura T. Xu J. Contractor A. (2014). The developmental switch in GABA polarity is delayed in fragile X mice. J. Neurosci. 34, 446–450. 10.1523/JNEUROSCI.4447-13.2014 24403144 PMC6608154

[B91] Hefti F. (1994). Neurotrophic factor therapy for nervous system degenerative diseases. J. Neurobiol. 25, 1418–1435. 10.1002/neu.480251109 7852995

[B92] Herbert K. M. Pimienta G. DeGregorio S. J. Alexandrov A. Steitz J. A. (2013). Phosphorylation of DGCR8 increases its intracellular stability and induces a progrowth miRNA Profile. Cell Rep. 5, 1070–1081. 10.1016/j.celrep.2013.10.017 24239349 PMC3892995

[B93] Hoeffer C. A. Sanchez E. Hagerman R. J. Mu Y. Nguyen D. V. Wong H.. (2012). Altered mTOR signaling and enhanced CYFIP2 expression levels in subjects with fragile X syndrome. Genes Brain Behav. 11, 332–341. 10.1111/j.1601-183X.2012.00768.x 22268788 PMC3319643

[B94] Horev G. Ellegood J. Lerch J. P. Son Y.-E. E. Muthuswamy L. Vogel H.. (2011). Dosage-dependent phenotypes in models of 16p11.2 lesions found in autism. Proc. Natl. Acad. Sci. U.S.A. 108, 17076–17081. 10.1073/pnas.1114042108 21969575 PMC3193230

[B95] Hori K. Nagai T. Shan W. Sakamoto A. Taya S. Hashimoto R.. (2014). Cytoskeletal regulation by AUTS2 in neuronal migration and neuritogenesis. Cell Rep. 9, 2166–2179. 10.1016/j.celrep.2014.11.045 25533347

[B96] Hsieh H.-L. Wang H.-H. Wu W.-B. Chu P.-J. Yang C.-M. (2010). Transforming growth factor-β1 induces matrix metalloproteinase-9 and cell migration in astrocytes: roles of ROS-dependent ERK- and JNK-NF-κB pathways. J. Neuroinflammation 7, 88. 10.1186/1742-2094-7-88 21134288 PMC3002339

[B97] Irwin S. A. Patel B. Idupulapati M. Harris J. B. Crisostomo R. A. Larsen B. P.. (2001). Abnormal dendritic spine characteristics in the temporal and visual cortices of patients with fragile-X syndrome: a quantitative examination. Am. J. Med. Genet. 98, 161–167. 10.1002/1096-8628(20010115)98:2<161::AID-AJMG1025>3.0.CO;2-B 11223852

[B98] Jacquemont S. Reymond A. Zufferey F. Harewood L. Walters R. G. Kutalik Z.. (2011). Mirror extreme BMI phenotypes associated with gene dosage at the chromosome 16p11.2 locus. Nature 478, 97–102. 10.1038/nature10406 21881559 PMC3637175

[B99] Jaworski J. Spangler S. Seeburg D. P. Hoogenraad C. C. Sheng M. (2005). Control of dendritic arborization by the phosphoinositide-3′-kinase-Akt-mammalian target of rapamycin pathway. J. Neurosci. 25, 11300–11312. 10.1523/JNEUROSCI.2270-05.2005 16339025 PMC6725892

[B100] Je H. S. Yang F. Ji Y. Nagappan G. Hempstead B. L. Lu B. (2012). Role of pro-brain-derived neurotrophic factor (proBDNF) to mature BDNF conversion in activity-dependent competition at developing neuromuscular synapses. Proc. Natl. Acad. Sci. U.S.A. 109, 15924–15929. 10.1073/pnas.1207767109 23019376 PMC3465384

[B101] Jellinger K. A. (2003). Rett syndrome - an update: review. J. Neural Transm. 110, 681–701. 10.1007/s00702-003-0822-z 12768363

[B102] Jeste S. S. Sahin M. Bolton P. Ploubidis G. B. Humphrey A. (2008). Characterization of autism in young children with tuberous sclerosis complex. J. Child Neurol. 23, 520–525. 10.1177/0883073807309788 18160549

[B103] Junger H. Junger W. G. (1998). CNTF and GDNF, but not NT-4, support corticospinal motor neuron growth via direct mechanisms. Neuroreport 9, 3749–3754. 10.1097/00001756-199811160-00033 9858391

[B104] Ka M. Condorelli G. Woodgett J. R. Kim W.-Y. (2014). mTOR regulates brain morphogenesis by mediating GSK3 signaling. Development 141, 4076–4086. 10.1242/dev.108282 25273085 PMC4302893

[B105] Kalscheuer V. M. FitzPatrick D. Tommerup N. Bugge M. Niebuhr E. Neumann L. M.. (2007). Mutations in autism susceptibility candidate 2 (AUTS2) in patients with mental retardation. Hum. Genet. 121, 501–509. 10.1007/s00439-006-0284-0 17211639

[B106] Karayiorgou M. Simon T. J. Gogos J. A. (2010). 22q11.2 microdeletions: linking DNA structural variation to brain dysfunction and schizophrenia. Nature Rev. Neurosci. 11, 402–416. 10.1038/nrn2841 20485365 PMC2977984

[B107] Kates W. R. Abrams M. T. Kaufmann W. E. Breiter S. N. Reiss A. L. (1997). Reliability and validity of MRI measurement of the amygdala and hippocampus in children with fragile X syndrome. Psychiatry Res. 75, 31–48. 10.1016/S0925-4927(97)00019-X 9287372

[B108] Keifer O. P. O'Connor D. M. Boulis N. M. (2014). Gene and protein therapies utilizing VEGF for ALS. Pharmacol. Therap. 141, 261–271. 10.1016/j.pharmthera.2013.10.009 24177067 PMC4659499

[B109] Keown C. Shih P. Nair A. Peterson N. Mulvey M. Müller R. A. (2013). Local functional overconnectivity in posterior brain regions is associated with symptom severity in autism spectrum disorders. Cell Rep. 5, 567–572. 10.1016/j.celrep.2013.10.003 24210815 PMC5708538

[B110] Klann E. Dever T. E. (2004). Biochemical mechanisms for translational regulation in synaptic plasticity. Nature Rev. Neurosci. 5, 931–942. 10.1038/nrn1557 15550948

[B111] Kwon C. H. Luikart B. W. Powell C. M. Zhou J. Matheny S. A. Zhang W.. (2006). Pten regulates neuronal arborization and social interaction in mice. Neuron 50, 377–388. 10.1016/j.neuron.2006.03.023 16675393 PMC3902853

[B112] Kwon C.-H. Zhu X. Zhang J. Baker S. J. (2003). mTor is required for hypertrophy of Pten-deficient neuronal soma *in vivo*. Proc. Natl. Acad. Sci. U.S.A. 100, 12923–12928. 10.1073/pnas.2132711100 14534328 PMC240720

[B113] Kwon C. H. Zhu X. Zhang J. Knoop L. L. Tharp R. Smeyne R. J.. (2001). Pten regulates neuronal soma size: a mouse model of Lhermitte-Duclos disease. Nat. Genet. 29, 404–411. 10.1038/ng781 11726927

[B114] Laplante M. Sabatini D. M. (2012). MTOR signaling in growth control and disease. Cell 149, 274–293. 10.1016/j.cell.2012.03.017 22500797 PMC3331679

[B115] Laviola L. Natalicchio A. Giorgino F. (2007). The IGF-I signaling pathway. Curr. Pharm. Des. 13, 663–669. 10.2174/138161207780249146 17346182

[B116] Leigh M. J. S. Nguyen D. V. Mu Y. Winarni T. I. Schneider A. Chechi T.. (2013). A randomized double-blind, placebo-controlled trial of minocycline in children and adolescents with fragile x syndrome. J. Dev. Behav. Pediatr. 34, 147–155. 10.1097/DBP.0b013e318287cd17 23572165 PMC3706260

[B117] Li Y. Wang H. Muffat J. Cheng A. W. Orlando D. A. Lovén J. Jaenisch R. (2013). Global transcriptional and translational repression in human-embryonic-stem-cell-derived Rett syndrome neurons. Cell Stem Cell 13, 446–458. 10.1016/j.stem.2013.09.001 24094325 PMC4053296

[B118] Liang P. Wan Y. Yan Y. Wang Y. Luo N. Deng Y.. (2010). MVP interacts with YPEL4 and inhibits YPEL4-mediated activities of the ERK signal pathway. Biochem. Cell Biol. 88, 445–450. 10.1139/O09-166 20555386

[B119] Louhivuori V. Vicario A. Uutela M. Rantamäki T. Louhivuori L. M. Castrén E.. (2011). BDNF and TrkB in neuronal differentiation of Fmr1-knockout mouse. Neurobiol. Dis. 41, 469–480. 10.1016/j.nbd.2010.10.018 21047554

[B120] Lu L. Hope B. T. Dempsey J. Liu S. Y. Bossert J. M. Shaham Y. (2005). Central amygdala ERK signaling pathway is critical to incubation of cocaine craving. Nat. Neurosci. 8, 212–219. 10.1038/nn1383 15657599

[B121] Lugtenberg D. Kleefstra T. Oudakker A. R. Nillesen W. M. Yntema H. G. Tzschach A.. (2009). Structural variation in Xq28: MECP2 duplications in 1% of patients with unexplained XLMR and in 2% of male patients with severe encephalopathy. Eur. J. Hum. Genet. 17, 444–453. 10.1038/ejhg.2008.208 18985075 PMC2986218

[B122] Luikenhuis S. Giacometti E. Beard C. F. Jaenisch R. (2004). Expression of MeCP2 in postmitotic neurons rescues Rett syndrome in mice. Proc. Natl. Acad. Sci. U.S.A. 101, 6033–6038. 10.1073/pnas.0401626101 15069197 PMC395918

[B123] Ma L. Chen Z. Erdjument-Bromage H. Tempst P. Pandolfi P. P. (2005). Phosphorylation and functional inactivation of TSC2 by Erk: implications for tuberous sclerosis and cancer pathogenesis. Cell 121, 179–193. 10.1016/j.cell.2005.02.031 15851026

[B124] Ma L. Teruya-Feldstein J. Bonner P. Bernardi R. Franz D. N. Witte D.. (2007). Identification of S664 TSC2 phosphorylation as a marker for extracellular signal-regulated kinase-mediated mTOR activation in tuberous sclerosis and human cancer. Cancer Res. 67, 7106–7112. 10.1158/0008-5472.CAN-06-4798 17671177

[B125] Ma X. M. Blenis J. (2009). Molecular mechanisms of mTOR-mediated translational control. Nat. Rev. Mol. Cell Biol. 10, 307–318. 10.1038/nrm2672 19339977

[B126] Malhotra D. Sebat J. (2012). CNVs: Harbingers of a rare variant revolution in psychiatric genetics. Cell 148, 1223–1241. 10.1016/j.cell.2012.02.039 22424231 PMC3351385

[B127] Marshall C. R. Noor A. Vincent J. B. Lionel A. C. Feuk L. Skaug J.. (2008). Structural variation of chromosomes in autism spectrum disorder. Am. J. Hum. Genet. 82, 477–488. 10.1016/j.ajhg.2007.12.009 18252227 PMC2426913

[B128] Martinowich K. Hattori D. Wu H. Fouse S. He F. Hu Y.. (2003). DNA methylation-related chromatin remodeling in activity-dependent BDNF gene regulation. Science 302, 890–893. 10.1126/science.1090842 14593184

[B129] Martinowich K. Manji H. Lu B. (2007). New insights into BDNF function in depression and anxiety. Nat. Neurosci. 10, 1089–1093. 10.1038/nn1971 17726474

[B130] Marui T. Hashimoto O. Nanba E. Kato C. Tochigi M. Umekage T.. (2004). Association between the neurofibromatosis-1 (NF1) locus and autism in the Japanese population. Am. J. Med. Genet. B Neuropsychiatr. Genet. 131B(1), 43–47. 10.1002/ajmg.b.20119 15389774

[B131] Matijevic T. Knezevic J. Slavica M. Pavelic J. (2009). Rett syndrome: from the gene to the disease. Eur. Neurol. 61, 3–10. 10.1159/000165342 18948693

[B132] McBride K. L. Varga E. A. Pastore M. T. Prior T. W. Manickam K. Atkin J. F.. (2010). Confirmation study of PTEN mutations among individuals with autism or developmental delays/mental retardation and macrocephaly. Autism Res. 3, 137–141. 10.1002/aur.132 20533527

[B133] McCarthy S. E. Makarov V. Kirov G. Addington A. M. McClellan J. Yoon S.. (2009). Microduplications of 16p11.2 are associated with schizophrenia. Nat. Genet. 41, 1223–1227. 10.1038/ng.474 19855392 PMC2951180

[B134] McGraw C. M. Samaco R. C. Zoghbi H. Y. (2011). Adult neural function requires MeCP2. Science 333, 186. 10.1126/science.1206593 21636743 PMC3150190

[B135] Meikle L. Talos D. M. Onda H. Pollizzi K. Rotenberg A. Sahin M.. (2007). A mouse model of tuberous sclerosis: neuronal loss of Tsc1 causes dysplastic and ectopic neurons, reduced myelination, seizure activity, and limited survival. J. Neurosci. 27, 5546–5558. 10.1523/JNEUROSCI.5540-06.2007 17522300 PMC6672762

[B136] Michalon A. Sidorov M. Ballard T. M. Ozmen L. Spooren W. Wettstein J. G.. (2012). Chronic pharmacological mGlu5 inhibition corrects Fragile X in adult mice. Neuron 74, 49–56. 10.1016/j.neuron.2012.03.009 22500629 PMC8822597

[B137] Mizoguchi T. Togawa S. Kawakami K. Itoh M. (2011). Neuron and sensory epithelial cell fate is sequentially determined by notch signaling in zebrafish lateral line development. J. Neurosci. 31, 15522–15530. 10.1523/JNEUROSCI.3948-11.2011 22031898 PMC6703510

[B138] Moreno-De-Luca D. Mulle J. G. Kaminsky E. B. Sanders S. J. Myers S. M. Adam M. P.. (2010). Deletion 17q12 is a recurrent copy number variant that confers high risk of autism and schizophrenia. Am. J. Hum. Genet. 87, 618–630. 10.1016/j.ajhg.2010.10.004 21055719 PMC2978962

[B139] Mukaddes N. M. Herguner S. (2007). Autistic disorder and 22q11.2 duplication. World J. Biol. Psychiatry 8, 127–130. 10.1080/15622970601026701 17455106

[B140] Mukai J. Dhilla A. Drew L. J. Stark K. L. Cao L. MacDermott A. B.. (2008). Palmitoylation-dependent neurodevelopmental deficits in a mouse model of 22q11 microdeletion. Nat. Neurosci. 11, 1302–1310. 10.1038/nn.2204 18836441 PMC2756760

[B141] Müller R. A. Shih P. Keehn B. Deyoe J. R. Leyden K. M. Shukla D. K. (2011). Underconnected, but how? A survey of functional connectivity MRI studies in autism spectrum disorders. Cereb. Cortex 21, 2233–2243. 10.1093/cercor/bhq296 21378114 PMC3169656

[B142] Murphy L. O. Blenis J. (2006). MAPK signal specificity: the right place at the right time. Trends Biochem. Sci. 31, 268–275. 10.1016/j.tibs.2006.03.009 16603362

[B143] Nishimura K. Nakamura K. Anitha A. Yamada K. Tsujii M. Iwayama Y.. (2007). Genetic analyses of the brain-derived neurotrophic factor (BDNF) gene in autism. Biochem. Biophys. Res. Commun. 356, 200–206. 10.1016/j.bbrc.2007.02.135 17349978

[B144] Nowicki S. T. Tassone F. Ono M. Y. Ferranti J. Croquette M. F. Goodlin-Jones B.. (2007). The Prader-Willi phenotype of fragile X syndrome. J. Dev. Behav. Pediatr. 28, 133–138. 10.1097/01.DBP.0000267563.18952.c9 17435464

[B145] O'Roak B. J. Vives L. Girirajan S. Karakoc E. Krumm N. Coe B. P.. (2012). Sporadic autism exomes reveal a highly interconnected protein network of de novo mutations. Nature 485, 246–250. 10.1038/nature10989 22495309 PMC3350576

[B146] Oksenberg N. Ahituv N. (2013). The role of AUTS2 in neurodevelopment and human evolution. Trends Genet. 29, 600–608. 10.1016/j.tig.2013.08.001 24008202 PMC3823538

[B147] Ola M. S. Nawaz M. I. Khan H. A. Alhomida A. S. (2013). Neurodegeneration and neuroprotection in diabetic retinopathy. Int. J. Mol. Sci. 14, 2559–2572. 10.3390/ijms14022559 23358247 PMC3588002

[B148] Osterweil E. K. Chuang S. C. Chubykin A. A. Sidorov M. Bianchi R. Wong R. K. S.. (2013). Lovastatin corrects excess protein synthesis and prevents epileptogenesis in a mouse model of Fragile X syndrome. Neuron 77, 243–250. 10.1016/j.neuron.2012.01.034 23352161 PMC3597444

[B149] Ouchi Y. Banno Y. Shimizu Y. Ando S. Hasegawa H. Adachi K.. (2013). Reduced adult hippocampal neurogenesis and working memory deficits in the Dgcr8-deficient mouse model of 22q11.2 deletion-associated schizophrenia can be rescued by IGF2. J. Neurosci. 33, 9408–9419. 10.1523/JNEUROSCI.2700-12.2013 23719809 PMC6618567

[B150] Pan F. Aldridge G. M. Greenough W. T. Gan W.-B. (2010). Dendritic spine instability and insensitivity to modulation by sensory experience in a mouse model of fragile X syndrome. Proc. Natl. Acad. Sci. U.S.A. 107, 17768–17773. 10.1073/pnas.1012496107 20861447 PMC2955121

[B151] Paribello C. Tao L. Folino A. Berry-Kravis E. Tranfaglia M. Ethell I. M.. (2010). Open-label add-on treatment trial of minocycline in fragile X syndrome. BMC Neurol. 10, 91. 10.1186/1471-2377-10-91 20937127 PMC2958860

[B152] Park H. Poo M. M. (2013). Neurotrophin regulation of neural circuit development and function. Nat. Rev. Neurosci. 14, 7–23. 10.1038/nrn3379 23254191

[B153] Penagarikano O. Mulle J. G. Warren S. T. (2007). The pathophysiology of fragile x syndrome. Annu. Rev. Genomics Hum. Genet. 8, 109–129. 10.1146/annurev.genom.8.080706.092249 17477822

[B154] Peprah E. (2012). Fragile X Syndrome: The FMR1 CGG Repeat Distribution Among World Populations. Ann. Hum. Genet. 76, 178–191. 10.1111/j.1469-1809.2011.00694.x 22188182 PMC3288311

[B155] Persico A. M. Bourgeron T. (2006). Searching for ways out of the autism maze: genetic, epigenetic and environmental clues. Trends Neurosci. 29, 349–358. 10.1016/j.tins.2006.05.010 16808981

[B156] Pinto D. Pagnamenta A. T. Klei L. Anney R. Merico D. Regan R.. (2010). Functional impact of global rare copy number variation in autism spectrum disorders. Nature 466, 368–372. 10.1038/nature09146 20531469 PMC3021798

[B157] Poo M. M. (2001). Neurotrophins as synaptic modulators. Nat. Rev. Neurosci. 2, 24–32. 10.1038/35049004 11253356

[B158] Portmann T. Yang M. Mao R. Panagiotakos G. Ellegood J. Dolen G.. (2014). Behavioral abnormalities and circuit defects in the basal ganglia of a mouse model of 16p11.2 deletion syndrome. Cell Rep. 7, 1077–1092. 10.1016/j.celrep.2014.03.036 24794428 PMC4251471

[B159] Pucilowska J. Vithayathil J. Tavares E. J. Kelly C. Karlo J. C. Landreth G. E. (2015). The 16p11.2 deletion mouse model of autism exhibits altered cortical progenitor proliferation and brain cytoarchitecture linked to the ERK MAPK pathway. J. Neurosci. 35, 3190–3200. 10.1523/JNEUROSCI.4864-13.2015 25698753 PMC6605601

[B160] Qureshi A. Y. Mueller S. Snyder A. Z. Mukherjee P. Berman J. I. Roberts T. P. L.. (2014). Opposing brain differences in 16p11.2 deletion and duplication carriers. J. Neurosci. 34, 11199–11211. 10.1523/JNEUROSCI.1366-14.2014 25143601 PMC4138332

[B161] Ramocki M. B. Tavyev Y. J. Peters S. U. (2010). The MECP2 duplication syndrome. Am. J. Med. Genet. A. 152A, 1079–1088. 10.1002/ajmg.a.33184 20425814 PMC2861792

[B162] Reichardt L. F. (2006). Neurotrophin-regulated signalling pathways. Philos. Trans. R. Soc. Lond. B Biol. Sci. 361, 1545–1564. 10.1098/rstb.2006.1894 16939974 PMC1664664

[B163] Reiss A. L. Faruque F. Naidu S. Abrams M. Beaty T. Bryan R. N.. (1993). Neuroanatomy of Rett syndrome: a volumetric imaging study. Ann. Neurol. 34, 227–234. 10.1002/ana.410340220 8338347

[B164] Ricceri L. De Filippis B. Laviola G. (2008). Mouse models of Rett syndrome: from behavioural phenotyping to preclinical evaluation of new therapeutic approaches. Behav. Pharmacol. 19, 501–517. 10.1097/FBP.0b013e32830c3645 18690105

[B165] Ronemus M. Iossifov I. Levy D. Wigler M. (2014). The role of de novo mutations in the genetics of autism spectrum disorders. Nat. Rev. Genet. 15, 133–141. 10.1038/nrg3585 24430941

[B166] Sadakata T. Furuichi T. (2009). Developmentally regulated Ca2+-dependent activator protein for secretion 2 (CAPS2) is involved in BDNF secretion and is associated with autism susceptibility. Cerebellum 8, 312–322. 10.1007/s12311-009-0097-5 19238500

[B167] Samuels I. S. Karlo J. C. Faruzzi A. N. Pickering K. Herrup K. Sweatt J. D.. (2008). Deletion of ERK2 mitogen-activated protein kinase identifies its key roles in cortical neurogenesis and cognitive function. J. Neurosci. 28, 6983–6995. 10.1523/JNEUROSCI.0679-08.2008 18596172 PMC4364995

[B168] Samuels I. S. Saitta S. C. Landreth G. E. (2009). MAP'ing CNS development and cognition: an ERKsome process. Neuron 61, 160–167. 10.1016/j.neuron.2009.01.001 19186160 PMC3663441

[B169] Santini E. Huynh T. N. MacAskill A. F. Carter A. G. Pierre P. Ruggero D.. (2013). Exaggerated translation causes synaptic and behavioural aberrations associated with autism. Nature 493, 411–415. 10.1038/nature11782 23263185 PMC3548017

[B170] Sato A. Kasai S. Kobayashi T. Takamatsu Y. Hino O. Ikeda K.. (2012). Rapamycin reverses impaired social interaction in mouse models of tuberous sclerosis complex. Nat. Commun. 3, 1292. 10.1038/ncomms2295 23250422 PMC3535343

[B171] Satoh Y. Kobayashi Y. Takeuchi A. Pagès G. Pouysségur J. Kazama T. (2011). Deletion of ERK1 and ERK2 in the CNS causes cortical abnormalities and neonatal lethality: Erk1 deficiency enhances the impairment of neurogenesis in Erk2-deficient mice. J. Neurosci. 31, 1149–1155. 10.1523/JNEUROSCI.2243-10.2011 21248139 PMC6632941

[B172] Schofield C. M. Hsu R. Barker A. J. Gertz C. C. Blelloch R. Ullian E. M. (2011). Monoallelic deletion of the microRNA biogenesis gene Dgcr8 produces deficits in the development of excitatory synaptic transmission in the prefrontal cortex. Neural Dev. 6:11. 10.1186/1749-8104-6-11 21466685 PMC3082233

[B173] Schüle B. Armstrong D. D. Vogel H. Oviedo A. Francke U. (2008). Severe congenital encephalopathy caused by MECP2 null mutations in males: central hypoxia and reduced neuronal dendritic structure. Clin. Genet. 74, 116–126. 10.1111/j.1399-0004.2008.01005.x 18477000

[B174] Sharma A. Hoeffer C. A. Takayasu Y. Miyawaki T. McBride S. M. Klann E.. (2010). Dysregulation of mTOR signaling in fragile X syndrome. J. Neurosci. 30, 694–702. 10.1523/JNEUROSCI.3696-09.2010 20071534 PMC3665010

[B175] Shi D. Xu S. Waddell J. Scafidi S. Roys S. Gullapalli R. P.. (2012). Longitudinal *in vivo* developmental changes of metabolites in the hippocampus of Fmr1 knockout mice. J. Neurochem. 123, 971–981. 10.1111/jnc.12048 23046047 PMC3957333

[B176] Shinawi M. Liu P. Kang S.-H. L. Shen J. Belmont J. W. Scott D. A.. (2010). Recurrent reciprocal 16p11.2 rearrangements associated with global developmental delay, behavioural problems, dysmorphism, epilepsy, and abnormal head size. J. Med. Genet. 47, 332–341. 10.1136/jmg.2009.073015 19914906 PMC3158566

[B177] Shiota C. Woo J. T. Lindner J. Shelton K. D. Magnuson M. A. (2006). Multiallelic disruption of the rictor gene in mice reveals that mTOR Complex 2 is essential for fetal growth and viability. Dev. Cell 11, 583–589. 10.1016/j.devcel.2006.08.013 16962829

[B178] Sidhu H. Dansie L. E. Hickmott P. W. Ethell D. W. Ethell I. M. (2014). Genetic removal of matrix metalloproteinase 9 rescues the symptoms of fragile X syndrome in a mouse model. J. Neurosci. 34, 9867–9879. 10.1523/JNEUROSCI.1162-14.2014 25057190 PMC4107404

[B179] Sigurdsson T. Stark K. L. Karayiorgou M. Gogos J. A. Gordon J. A. (2010). Impaired hippocampal-prefrontal synchrony in a genetic mouse model of schizophrenia. Nature 464, 763–767. 10.1038/nature08855 20360742 PMC2864584

[B180] Slegtenhorst-Eegdeman K. E. de Rooij D. G. Verhoef-Post M. van de Kant H. J. Bakker C. E. Oostra B. A.. (1998). Macroorchidism in FMR1 knockout mice is caused by increased Sertoli cell proliferation during testicular development. Endocrinology 139, 156–162. 10.1210/en.139.1.156 9421410

[B181] Spence S. J. Cantor R. M. Chung L. Kim S. Geschwind D. H. Alarcón M. (2006). Stratification based on language-related endophenotypes in autism: attempt to replicate reported linkage. Am. J. Med. Genet. B Neuropsychiatr. Genet. 141, 591–598. 10.1002/ajmg.b.30329 16752361 PMC3653581

[B182] Sultana R. Yu C.-E. Yu J. Munson J. Chen D. Hua W.. (2002). Identification of a novel gene on chromosome 7q11.2 interrupted by a translocation breakpoint in a pair of autistic twins. Genomics 80, 129–134. 10.1006/geno.2002.6810 12160723

[B183] Supekar K. Uddin L. Q. Khouzam A. Phillips J. Gaillard W. D. Kenworthy L. E.. (2013). Brain Hyperconnectivity in Children with Autism and its Links to Social Deficits. Cell Rep. 5, 738–747. 10.1016/j.celrep.2013.10.001 24210821 PMC3894787

[B184] Sweatt J. D. (2004). Mitogen-activated protein kinases in synaptic plasticity and memory. Curr. Opin. Neurobiol. 14, 311–317. 10.1016/j.conb.2004.04.001 15194111

[B185] Takei N. Nawa H. (2014). mTOR signaling and its roles in normal and abnormal brain development. Front. Mol. Neurosci. 7:28. 10.3389/fnmol.2014.00028 24795562 PMC4005960

[B186] Tang G. Gudsnuk K. Kuo S. H. Cotrina M. L. Rosoklija G. Sosunov A.. (2014). Loss of mTOR-dependent macroautophagy causes autistic-like synaptic pruning deficits. Neuron 83, 1131–1143. 10.1016/j.neuron.2014.07.040 25155956 PMC4159743

[B187] Thomanetz V. Angliker N. Cloëtta D. Lustenberger R. M. Schweighauser M. Oliveri F.. (2013). Ablation of the mTORC2 component rictor in brain or Purkinje cells affects size and neuron morphology. J. Cell Biol. 201, 293–308. 10.1083/jcb.201205030 23569215 PMC3628512

[B188] Tian D. Stoppel L. J. Heynen A. J. Lindemann L. Jaeschke G. Mills A. A.. (2015). Contribution of mGluR5 to pathophysiology in a mouse model of human chromosome 16p11.2 microdeletion. Nat. Neurosci. 18, 182–184. 10.1038/nn.3911 25581360 PMC4323380

[B189] Tsai S. J. (2005). Is autism caused by early hyperactivity of brain-derived neurotrophic factor? Med. Hypotheses 65, 79–82. 10.1016/j.mehy.2005.01.034 15893122

[B190] Uutela M. Lindholm J. Louhivuori V. Wei H. Louhivuori L. M. Pertovaara A.. (2012). Reduction of BDNF expression in Fmr1 knockout mice worsens cognitive deficits but improves hyperactivity and sensorimotor deficits. Genes Brain Behav. 11, 513–523. 10.1111/j.1601-183X.2012.00784.x 22435671

[B191] Van Bon B. W. M. Coe B. P. Bernier R. Green C. Gerdts J. Witherspoon K.. (2015). Disruptive de novo mutations of DYRK1A lead to a syndromic form of autism and ID. Mol. Psychiatry. 10.1038/mp.2015.5. [Epub ahead of print]. 25707398 PMC4547916

[B193] Varga E. A. Pastore M. Prior T. Herman G. E. McBride K. L. (2009). The prevalence of PTEN mutations in a clinical pediatric cohort with autism spectrum disorders, developmental delay, and macrocephaly. Genet. Med. 11, 111–117. 10.1097/GIM.0b013e31818fd762 19265751

[B194] Vissers M. E. Cohen M. X. Geurts H. M. (2012). Brain connectivity and high functioning autism: a promising path of research that needs refined models, methodological convergence, and stronger behavioral links. Neurosci. Biobehav. Rev. 36, 604–625. 10.1016/j.neubiorev.2011.09.003 21963441

[B195] Walters R. G. Jacquemont S. Valsesia A. de Smith A. J. Martinet D. Andersson J.. (2010). A new highly penetrant form of obesity due to deletions on chromosome 16p11.2. Nature 463, 671–675. 10.1038/nature08727 20130649 PMC2880448

[B196] Wang X. Snape M. Klann E. Stone J. G. Singh A. Petersen R. B.. (2012). Activation of the extracellular signal-regulated kinase pathway contributes to the behavioral deficit of fragile x-syndrome. J. Neurochem. 121, 672–679. 10.1111/j.1471-4159.2012.07722.x 22393900

[B197] Weiss L. A. Shen Y. Korn J. M. Arking D. E. Miller D. T. Fossdal R.. (2008). Association between microdeletion and microduplication at 16p11.2 and autism. N. Engl. J. Med. 358, 667–675. 10.1056/NEJMoa075974 18184952

[B198] Weissmiller A. M. Wu C. (2012). Current advances in using neurotrophic factors to treat neurodegenerative disorders. Transl. Neurodegener. 1:14. 10.1186/2047-9158-1-14 23210531 PMC3542569

[B199] Worby C. A. Dixon J. E. (2014). PTEN. Annu. Rev. Biochem. 83, 641–669. 10.1146/annurev-biochem-082411-113907 24905788

[B200] Wu G. Y. Deisseroth K. Tsien R. W. (2001). Spaced stimuli stabilize MAPK pathway activation and its effects on dendritic morphology. Nat. Neurosci. 4, 151–158. 10.1038/83976 11175875

[B201] Xu B. Karayiorgou M. Gogos J. A. (2010). MicroRNAs in psychiatric and neurodevelopmental disorders. Brain Res. 1338, 78–88. 10.1016/j.brainres.2010.03.109 20388499 PMC2883644

[B202] Yagi H. Furutani Y. Hamada H. Sasaki T. Asakawa S. Minoshima S.. (2003). Role of TBX1 in human del22q11.2 syndrome. Lancet 362, 1366–1373. 10.1016/S0140-6736(03)14632-6 14585638

[B203] Yuan T. L. Cantley L. C. (2008). PI3K pathway alterations in cancer: variations on a theme. Oncogene 27, 5497–5510. 10.1038/onc.2008.245 18794884 PMC3398461

[B204] Yufune S. Satoh Y. Takamatsu I. Ohta H. Kobayashi Y. Takaenoki Y.. (2015). Transient blockade of ERK phosphorylation in the critical period causes autistic phenotypes as an adult in mice. Sci. Rep. 5:10252. 10.1038/srep10252 25993696 PMC4438718

[B205] Zhang H. Cicchetti G. Onda H. Koon H. B. Asrican K. Bajraszewski N.. (2003). Loss of Tsc1/Tsc2 activates mTOR and disrupts PI3K-Akt signaling through downregulation of PDGFR. J. Clin. Invest. 112, 1223–1233. 10.1172/JCI200317222 14561707 PMC213485

[B206] Zhou J. Blundell J. Ogawa S. Kwon C.-H. Zhang W. Sinton C.. (2009). Pharmacological inhibition of mTORC1 suppresses anatomical, cellular, and behavioral abnormalities in neural-specific Pten knock-out mice. J. Neurosci. 29, 1773–1783. 10.1523/JNEUROSCI.5685-08.2009 19211884 PMC3904448

[B207] Zhou J. Parada L. F. (2012). PTEN signaling in autism spectrum disorders. Curr. Opin. Neurobiol. 22, 873–879. 10.1016/j.conb.2012.05.004 22664040

[B208] Zhou Z. Hong E. J. Cohen S. Zhao W. Ho H. Y. Schmidt L.. (2006). Brain-specific phosphorylation of MeCP2 regulates activity-dependent Bdnf transcription, dendritic growth, and spine maturation. Neuron 52, 255–269. 10.1016/j.neuron.2006.09.037 17046689 PMC3962021

[B209] Zoghbi H. Y. Bear M. F. (2012). Synaptic dysfunction in neurodevelopmental disorders associated with autism and intellectual disabilities. Cold Spring Harb. Perspect. Biol. 4:a009886. 10.1101/cshperspect.a009886 22258914 PMC3282414

[B210] Zoncu R. Efeyan A. Sabatini D. M. (2011). mTOR: from growth signal integration to cancer, diabetes and ageing. Nat. Rev. Mol. Cell Biol. 12, 21–35. 10.1038/nrm3025 21157483 PMC3390257

[B211] Zuccato C. Cattaneo E. (2009). Brain-derived neurotrophic factor in neurodegenerative diseases. Nat. Rev. Neurol. 5, 311–322. 10.1038/nrneurol.2009.54 19498435

[B212] Zufferey F. Sherr E. H. Beckmann N. D. Hanson E. Maillard A. M. Hippolyte L.. (2012). A 600 kb deletion syndrome at 16p11.2 leads to energy imbalance and neuropsychiatric disorders. J. Med. Genet. 49, 660–668. 10.1136/jmedgenet-2012-101203 23054248 PMC3494011

